# Mpath maps multi-branching single-cell trajectories revealing progenitor cell progression during development

**DOI:** 10.1038/ncomms11988

**Published:** 2016-06-30

**Authors:** Jinmiao Chen, Andreas Schlitzer, Svetoslav Chakarov, Florent Ginhoux, Michael Poidinger

**Affiliations:** 1 Singapore Immunology Network (SIgN), Agency for Science, Technology and Research (A*STAR), 8A Biomedical Grove, #03-06, Singapore 138648, Singapore

## Abstract

Single-cell RNA-sequencing offers unprecedented resolution of the continuum of state transition during cell differentiation and development. However, tools for constructing multi-branching cell lineages from single-cell data are limited. Here we present Mpath, an algorithm that derives multi-branching developmental trajectories using neighborhood-based cell state transitions. Applied to mouse conventional dendritic cell (cDC) progenitors, Mpath constructs multi-branching trajectories spanning from macrophage/DC progenitors through common DC progenitor to pre-dendritic cells (preDC). The Mpath-generated trajectories detect a branching event at the preDC stage revealing preDC subsets that are exclusively committed to cDC1 or cDC2 lineages. Reordering cells along cDC development reveals sequential waves of gene regulation and temporal coupling between cell cycle and cDC differentiation. Applied to human myoblasts, Mpath recapitulates the time course of myoblast differentiation and isolates a branch of non-muscle cells involved in the differentiation. Our study shows that Mpath is a useful tool for constructing cell lineages from single-cell data.

## Abstract (web summary)

The tools for constructing cell lineages from single cell data are limited. Here, the authors present Mpath: an algorithm to derive multi-branching trajectories using neighbourhood-based cell state transitions, and use this to predict developmental trajectories in dendritic and muscle cells.

## Graphical abstract

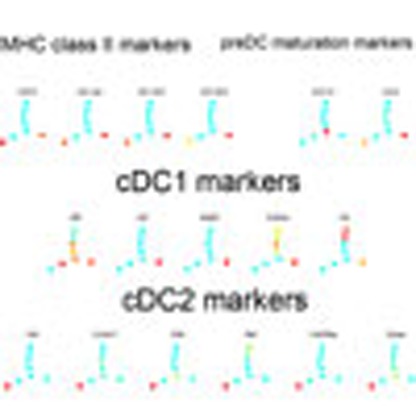

Single-cell sequencing is a relatively recent technique that offers unprecedented insights into the functionality and development of complex cell lineages^1^,^2^,^3^,^4^,^5^,^6^,^7^,^8^,^9^,^10^,^11^,^12^,^13^. In particular, this technique has revealed that a seemingly homogenous cell population often comprises cells at various proliferating and differentiating stages^2^,^8^,^9^,^10^. Furthermore, a continuum of transitional cell states has been found to constitute the progression between discrete states^5^,^11^. However, tools and methodology for constructing cell lineages from single-cell data are few, and have some limitations. The NBOR algorithm (‘neighborhood-based ordering of single cells'), a method we recently developed, leverages the continuum of transitional cell states to reconstruct dendritic cell (DC) progenitor development lineage^9^. However, NBOR assumes the developmental trajectory is non-branching, and hence works optimally for linear development lineage with no branching. Several other methods that allow for branching have been recently proposed to enable the analysis of more complex system. Diffusion map was recently adapted for dimensionality reduction of single-cell data, and was shown to outperform principal component analysis (PCA) and t-distributed stochastic neighbor embedding for detecting branching developmental trajectories from massive quantitative PCR or RNA-seq data^11^,^14^. However, the performance of diffusion map can be hampered by low number of cells, especially when data are generated by RNA-sequencing^14^. Another method named single-cell clustering using bifurcation analysis (SCUBA) detects branching events of development via investigating dynamic changes of gene expression pattern using bifurcation theory^15^. However, it assumes every branching event gives rise to only two lineages and requires time-course data sampled with sufficient temporal resolution. One other method named monocle^10^ also produces multi-branching trajectories of cells' progress through differentiation. The algorithm first represents each cell as a point in a high-dimensional Euclidean space, and then reduces the dimensionality using independent component analysis. In the low-dimensional space, monocle constructs a minimum spanning tree (MST) to connect the cells, and identifies the longest backbone path through the MST. However, with the latest single-cell RNA-seq technologies measuring thousands and even tens of thousands of cells, MSTs connecting a large number of cells become complex and difficult to interpret.

To overcome the limitations of existing methods, we here propose a novel algorithm termed Mpath for constructing multi-branching single-cell trajectories of cellular state transition from single-cell RNA-seq data. Mpath is flexible in identifying both linear and branching development pathways. It does not require massive number of cells or time-course data. Furthermore, it can infer progenitor stage progression and identify subset-committed progenitor cells using only signature genes derived from comparing end stages of differentiated cell subsets. We show the utility of this algorithm on our recently published conventional dendritic cell (cDC)^9^ and publicly available human myoblast data sets^10^. Using these data sets, we show that Mpath produces more biologically relevant results as compared with existing methods. And it is the only method that faithfully recapitulates previously published experimental data of cDC development in particular the exclusive cDC subset-commitment of cDC progenitors.

## Results

### General framework of Mpath

**Figure 1:**
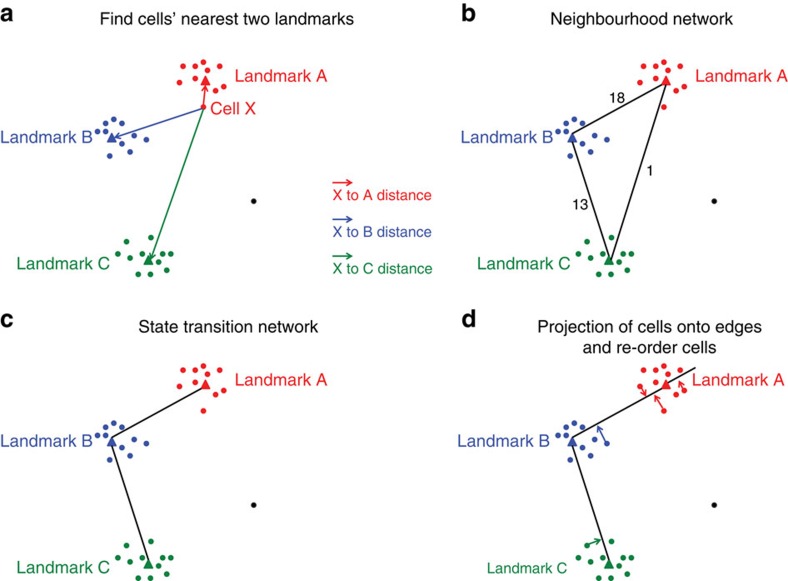
A two-dimensional visual example of Mpath algorithm. (**a**) Identify the nearest two landmarks for every cell. (**b**) Build neighbourhood network to connect the landmarks. Numbers on edges indicate the number of cells for which the two landmarks on both ends of the edge are the nearest neighbouring landmarks. (**c**) State transition network after trimming edges with lower cell count support. (**d**) Project individual cells onto the edges and re-order the cells according to the position of projection points.

As illustrated in the flow chart (Supplementary Fig. 1), Mpath first clustered the cells and designated landmark clusters with each representing discrete cell states. When applied to a set of single-cell data comprising known progenitor states (such as with a time course or through fluorescence activated cell sorting (FACS) sorting), landmark clusters comprise cells mainly from one population (as defined by the time points or the FACS sorting). It subsequently identified cells that were potentially transitioning between landmark states based on their similarities in transcriptional profiles shared with both end states. The count of such cells was then used to estimate the likelihood of the state transition being true. A pair of landmark states with high count of such cells was likely two adjacent stages along the developmental lineage. Mpath then built a neighbourhood network of landmarks in which edges connecting landmarks were weighted by the number of cells that were at transitional stage. As edges of low weights were likely artifacts owing to noise, Mpath removed edges with low cell count support using a heuristic approach, giving rise to a trimmed network that allows connecting all landmarks by including the minimal number of edges and the maximum number of cells on edges. The trimmed network reconstructed a multi-branching state transition network of cellular development and differentiation. Lastly, Mpath placed individual cells in order along the developmental trajectories between every pair of neighbouring landmark states. A two-dimensional visual example of the algorithm for going from a point cloud to a state transition graph and cell re-ordering is illustrated in Fig. 1. Detailed description of Mpath is given in the Methods section. Next, we applied Mpath to two single-cell differentiation data sets of biological relevance including cDC (ref. 9) and human myoblasts^10^.

### Mpath constructed multi-branching dendritic cell lineages

**Figure 2:**
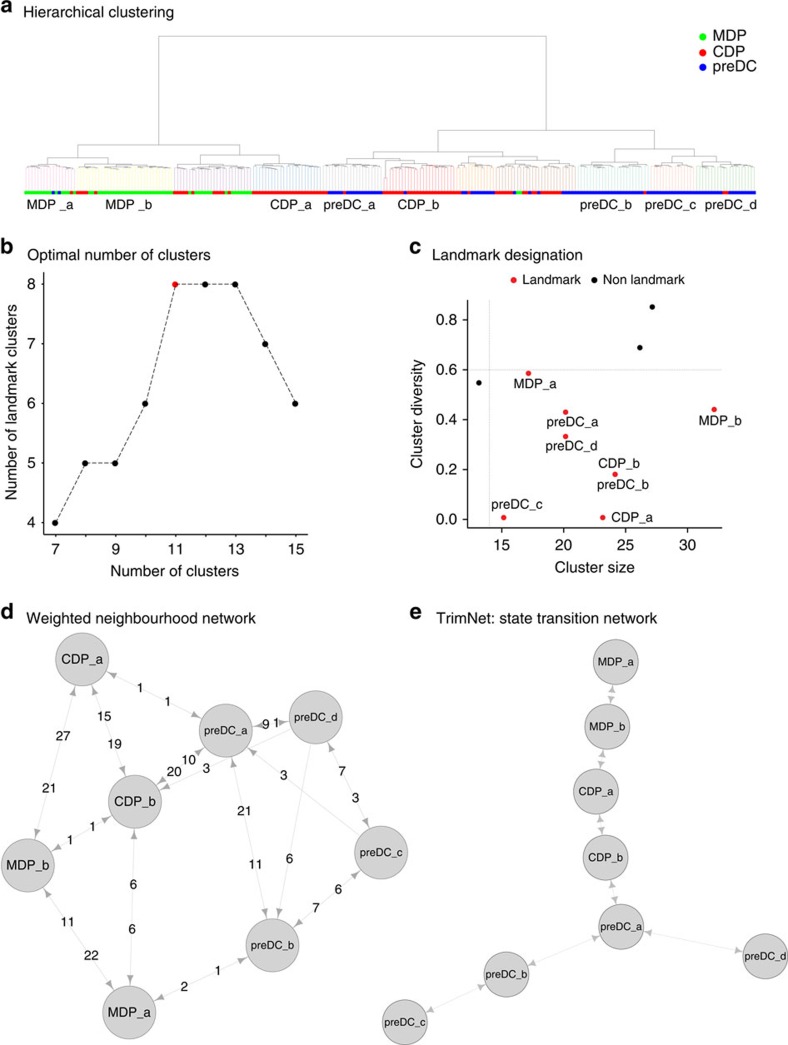
Mpath constructed multi-branching dendritic cell (DC) lineages. Mpath was applied to single-cell RNA-sequencing data of mouse bone marrow-derived MDP, CDP and preDC cells. (**a**) Hierarchical clustering of cells using Euclidean distance and Ward.D agglomeration method. The clusters were defined by cutting the dendrogram into 11 branches. Clusters comprising cells mainly from one population were named by the major cell type of the respective clusters. (**b**) Determine the optimal number of clusters by examining the plot of landmark numbers versus total number of clusters. (**c**) Clusters that passed both size and purity cutoff were assigned as landmark clusters. (**d**) Weighted neighbourhood network of landmarks. Nodes represent landmarks; edges represent putative transitions between landmark states; numbers on edges represent edge weights measured by number of cells that were located in the neighbourhood between the landmarks. (**e**) State transition network after trimming low-weighted edges in the weighted neighbourhood network.

Murine cDCs are constituted of two main lineages, namely cDC1 and cDC2 (refs 16, 17), each controlled by distinct transcriptional programs which start with the macrophage and dendritic cell progenitor (MDP), followed by the common DC progenitor (CDP) and then pre-dendritic cell (preDC), which migrate to peripheral organs and terminally differentiate into cDC1 and cDC2 subsets^9^. In our recent study, we isolated all mouse bone marrow DC progenitors, including 59 Lin^−^CD11c^−^MHCII^−^CD135^+^CSF-1R^+^CD117^hi^ MDPs, 96 Lin^−^CD11c^−^MHCII^−^CD135^+^CSF-1R^+^CD117^int^ CDPs and 96 Lin^−^CD11c^+^MHCII^−^CD135^+^CD172α^−^ preDCs, by flow cytometry and assessed their single-cell transcriptome by microfluidic single-cell messenger RNA sequencing^9^. Using this approach, we showed that early lineage-priming occurs in the bone marrow at the CDP level and identified cDC1 and cDC2-committed preDCs in the bone marrow^9^. Here, using Mpath analysis, we confirm and extend our previous findings. Mpath was applied using mature cDC1 versus cDC2 signature genes derived from microarray analysis of FACS sorted splenic cDCs (GSE60782). As described in the landmark designation step of Mpath methodology, Mpath clustered cells using these signature genes (Fig. 2a) and identified the inflexion point of number of landmarks versus number of clusters to be 11 (Fig. 2b). Among the total 11 clusters, 8 landmark clusters were designated (Fig. 2c). Next Mpath reconstructed a state transition network to infer putative transitions between the landmark states (Fig. 2d,e). The resulting state transition network (Fig. 2e) recapitulated the known cascade starting with MDP, then CDP and lastly preDC. However, according to the state transition network, Mpath identified different stages of DC progenitors. Landmark MDP_a and MDP_b were early and late MDP states, respectively; landmark CDP_a and CDP_b were early and late CDP states, respectively. Notably, it detected a branching event at the early preDC stage revealing divergent cell fates of single preDCs. Two separate lineages branched out from common early preDCs giving rise to distinct late preDC populations which potentially constitute immediate precursors of cDC1 and cDC2 subsets as recently published^9^.

### Mpath-derived DC lineages confirm early DC subset commitment

**Figure 3:**
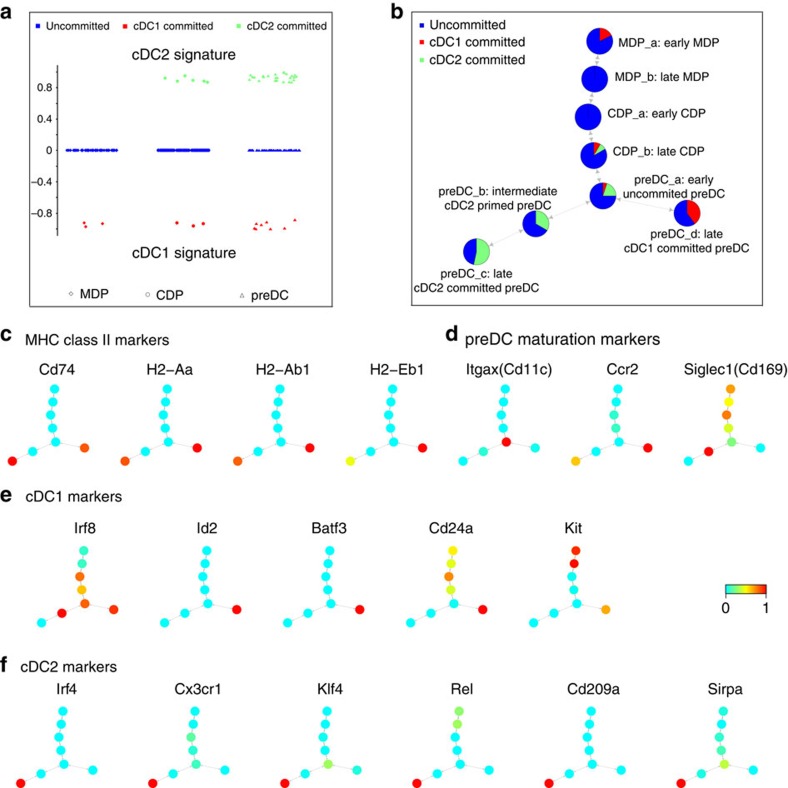
Mpath-derived dendritic cell lineages confirm early DC subset commitment. We annotated each landmark state by cMAP analysis and examining progression of DEGs. (**a**) cMAP analysis of single cells using mature cDC2 versus cDC1 signature genes. *Y* axis represents cMAP scores of individual cells; positive cMAP scores indicate enrichment of cDC2 signature; negative cMAP scores indicate enrichment of cDC1 signature; zero cMAP score indicates null enrichment. (**b**) Proportion of cDC1-committed, cDC2-committed and uncommitted cells at each landmark state of the state transition network. Uncommitted, cDC1-committed and cDC2-committed cells were defined by cMAP analysis as shown in **a**. (**c**) Overlay of MHC class II marker expression on the state transition network. (**d**) Overlay of preDC maturation marker expression on the state transition network. (**e**) Overlay of cDC1 marker expression on the state transition network. (**f**) Overlay of cDC2 marker expression on the state transition network. In (**c**–**f**), the nodes are colour-coded by TPM values scaled to range [0, 1].

To further delineate the Mpath-derived state transition network, we performed connectivity map (cMAP)^18^ analysis using mature cDC1 versus cDC2 signature genes to identify putative cDC subset-primed cells at MDP, CDP and preDC stages. Similar to gene set enrichment analysis, cMAP is a pattern-matching tool that can be used to detect similarities between cells and gene signatures. cMAP analysis suggested priming towards the cDC1 lineage can potentially occur as early as MDP stage, whereas priming towards the cDC2 lineage was only detected from CDP stage onwards (Fig. 3a). The number of subset-primed cells increased along cell maturation from MDP through CDP to preDC (Fig. 3a). The ratio of cDC1-committed to cDC2-commited cells is in agreement with the notion that cDC2 cells are more abundant in number compared with cDC1 cells^19^. Tracking the changes in the proportion of subset-committed cells through the state transition network revealed that majority of the cells was uncommitted before the branching point (Fig. 3b). A minority of late CDPs and early preDCs were either committed to cDC1 or cDC2, implying dual subset potentials at late CDP and early preDC stages. From the branching point onwards, the proportion of subset-committed cells substantially increased and the dual subset potential was lost. The branch spanning preDC_a, preDC_b and preDC_c was exclusively committed to cDC2 while the branch spanning preDC_a and preDC_d was exclusively committed to cDC1. Based on these observations, we annotated the state transition network for its biological relevance (Fig. 3b).

To further verify the annotation of the state transition network, we derived signature genes of preDC_a, preDC_c and preDC_d landmarks via ANOVA differential expression analysis (Supplementary Fig. 2a, Supplementary Data 1). Gene ontology analysis of these ANOVA derived genes revealed that along the development from preDC_a to preDC_c or preDC_d, biological processes associated with cell cycle and proliferation decreased, whilst immune related functions such as antigen presentation, immune response and cell activation increased (Supplementary Fig. 2b,c). Major histocompatibility complex (MHC) class II molecules (*H2-Aa, H2-Ab1* and *H2-Eb1*) and associated molecule *Cd74* were upregulated only at preDC_c and preDC_d, the two end stages of preDC differentiation (Fig. 3c). The bone marrow egression marker *Ccr2* was highly expressed in both preDC_c and preDC_d, implying these cells were about to migrate from bone marrow to blood (Fig. 3d). In addition, *Ly6d*, *Siglech* and *Cd11c* were significantly (adjusted *P* value<0.05, ANOVA) downregulated in preDC_c and preDC_d compared with preDC_a (Supplementary Data 1). The expression of higher levels of *MHC-II* and *Ccr2* together with lower levels of *Ly6d*, *Siglech* and *Cd11c* are known hallmarks of DC maturation and differentiation^9^,^20^. As such, our analysis suggests preDC_a represents an early preDC state having a proliferative signature in addition to being less differentiated; preDC_c and preDC_d represent further differentiated cell states with lower capacity of proliferation but transcriptomic commitment to either cDC1 or cDC2. Our findings implied that a switch from cell cycle and proliferation to cell differentiation occurred during cDC maturation and development. This proliferation to differentiation switch was also observed at the CDP stage as landmark CDP_b downregulated cell cycle genes compared with landmark CDP_a (Supplementary Table 1). Our observations are in agreement with earlier findings where DC progenitors were shown to downregulate their proliferation activity on differentiation^9^,^20^.

Previously reported cDC1 master regulators (*Id2* (ref. 19), *Batf3* (refs 21, 22)) were only upregulated in landmark preDC_d (Fig. 3e). Another well-studied cDC1 marker, *Irf8* (ref. 19), was found to be upregulated starting from early CDP stage then downregulated in preDC_c but not preDC_d (Fig. 3e). Recently described cDC1 early markers, *Kit* (refs 22, 23) and *Cd24α* (refs 9, 23, 24), were upregulated in preDC_d but not in preDC_a, preDC_b or preDC_c (Fig. 3e). Other markers for which there is some evidence for their involvement in cDC1 development (*Tlr3* (ref. 23), *Ifi205* (ref. 23), *Ncf4* (ref. 23) were also significantly (adjusted *P* value<0.05, ANOVA) upregulated in preDC_d compared with preDC_c (Supplementary Data 1). On the other hand, previously reported cDC2-specific markers and dominant transcription factors (*Irf4* (refs 25, 26), *Cx3cr1* (ref. 19), *Klf4* (ref. 27), *Rel* (ref. 28), *Cd209a* (ref. 25) and *Sirpa* (ref. 24) were only upregulated in landmark preDC_c (Fig. 3f, Supplementary Data 1). The progression of these marker expression provided further evidence that preDC_d and preDC_c were committed to cDC1 and cDC2, respectively.

In addition to the previously described markers, our analysis identified new putative markers for early uncommitted, cDC1-committed and cDC2-committed preDCs (Supplementary Fig. 2a, Supplementary Data 1). Both cDC1- and cDC2-committed preDCs upregulated transcriptional co-activator *CIITA* (Supplementary Fig. 2d) that was reported as the master regulator of *MHC* class II genes^29^. Moreover, the up-regulation of *CIITA* paralleled that of major MHC class II molecules (*H2-Aa, H2-Ab1* and *H2-Eb1*) and associated molecule *Cd74* (Supplementary Fig. 2d, Fig. 3c)*. CIITA* has been referred to as the master control factor for the expression of MHC class II genes^29^. And it has been shown to exhibit differentiation-stage-specific pattern of expression that precisely parallels that of MHCII genes^30^. Our Mpath analysis implies *CIITA* is responsible for driving the activation of MHCII genes during DC early development in the bone marrow. In addition, transcriptional factor *Irf5* was also found to be upregulated in both subset-committed preDCs (Supplementary Fig. 2d). Novel putative transcriptional regulators of pre-cDC1 included *Ifi205* and *Pbx1* that were recently described in our previous study^9^, as well as *Lbh*, *Mnda* and *Rbbp7* (Supplementary Fig. 2e). Their involvement in cDC1 development remains unknown. Compared with cDC1, cDC2 comprises more heterogeneous cell populations and its development is less understood. We identified several putative early regulators for cDC2 lineage, including *Atf3*, *Fosb*, *Ifi204*, *Irf7*, *Junb*, *Mphosph8*, *Nfkbiz*, *Pfdn5*, *Zeb2* and *Zfp36* (Supplementary Fig. 2f). Their roles in cDC2 commitment remain to be investigated. Besides transcriptional regulators, other types of putative markers were also identified (Supplementary Data 1).

Guided by a reference data set of mature cDC1 and cDC2, Mpath was able to accurately recapitulate the progression from MDP through CDP to preDC, at the same time capture both the proliferation to differentiation switch and cell marker progression, and elucidated the branching event that led to exclusive commitment to cDC1 or cDC2 lineages. This in depth delineation of the transcriptomic events leading to terminal DC differentiation illustrates the power of Mpath.

### Mpath re-ordered cells and revealed waves of gene regulation

**Figure 4:**
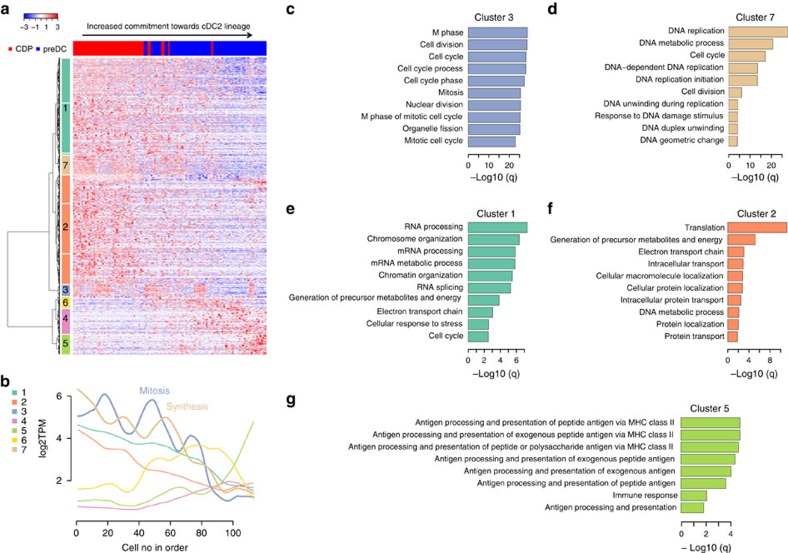
Mpath re-ordered single cells and revealed sequential waves of gene regulation during DC subset commitment. Mpath re-ordered single cells along the developmental trajectory of cDC2 lineage commitment. (**a**) Heatmap and hierarchical clustering of genes that were differentially expressed along the developmental trajectory from CDP to cDC2-committed preDCs (genes in rows and cells in columns). Cells were placed in the order of increased commitment towards cDC2 lineage. Clustering of genes identified seven distinct groups. (**b**) Average expression per group was smoothed by loess regression and plotted in the line chart. (**c**–**g**) Gene ontology analysis identified biological processes significantly (adjusted *P* value<0.05, DAVID gene ontology analysis) enriched by genes from group 3 (**c**), group 7 (**d**), group 1 (**e**), group 2 (**f**) and group 5 (**g**). The side bar of **a**, lines of **b**, bar charts of **c**–**g** are colour-coded by gene clusters.

Mpath re-ordered individual cells along DC developmental trajectories to resemble pseudo-temporal kinetics of gene expression during differentiation. It then identified genes whose expression changed significantly (adjusted *P* value<0.05, likelihood ratio test) as a function of the pseudo-temporal ordering using generalized additive models (GAMs)^31^. Finally, it performed cluster analysis on the differentially regulated genes to classify them based on pseudo-temporal expression patterns. This analysis identified seven distinct clusters of genes that were regulated differentially during development from CDP to cDC2-committed preDC (Fig. 4a, Supplementary Data 2). Clusters 3 and 7 included genes mainly involved in mitosis and synthesis phases of cell cycle respectively (Fig. 4b–d). Expression of these genes showed an overall downward trend, suggesting DC progenitor decreased cell cycle and proliferation as they became more differentiated to the late subset-committed preDC stage. It is expected that CDPs and early uncommitted preDCs were less differentiated and in a more proliferative state as compared with the more differentiated late subset-committed preDCs. Notably, expression of the mitosis and synthesis genes followed two nearly out-of-phase waves with respect to the pseudo-temporal order. When mitosis genes were upregulated, synthesis genes were downregulated and vice versus. It appears that this lineage undergoes several cell-cycle peaks during DC maturation. Cluster 1 comprised genes that were first highly expressed in CDPs and then gradually downregulated. These genes were significantly (adjusted *P* value<0.05, DAVID gene ontology analysis) enriched for biological processes central to RNA processing, chromosome/chromatin organization and metabolism (Fig. 4e). In particular, expression of genes associated with chromosome/chromatin organization shows similar trends as cell cycle genes, suggesting that the remodeling of chromatin occurs during cell cycle. Chromatin remodelling is known to be crucial during development^32^ and haematopoietic differentiation^33^. Mpath analysis showed a temporal coupling among cell cycle, chromatin organization and cell differentiation, implying these processes are interrelated during DC differentiation. Cluster 2 included genes that were highly expressed in CDPs and rapidly downregulated in early preDCs. These genes were highly enriched for biological processes central to translation, protein transport and metabolism (Fig. 4f). The differentiation of monocytes into DCs and the development of DCs in lymphoid organs and peripheral tissues have been shown to be dependent on metabolic pathways^34^,^35^. However little is known about the metabolism of CDPs or MDPs. Our analysis implies the role of metabolism during DC development from bone marrow-derived precursors. Cluster 6, including genes such as *Spib*, *Tcf4*, *Siglech*, *Ly6d* and *Itgax*, was first upregulated on transition from CDP to preDC and then downregulated as preDCs became more differentiated towards the cDC2 lineage. Cluster 4 constituted a second wave of gene activation occurring at a later time point on the transition from early preDC to late cDC2-committed preDC. During the last wave of transcriptional changes, genes from cluster 5 were sharply upregulated in the cDC2-committed preDCs. These genes were enriched for biological processes central to antigen processing and presentation via MHC class II (Fig. 4g) which marks further maturation and differentiation of preDCs. Similarly, during development from CDP to cDC1-committed preDC, we identified six distinct clusters of differentially expressed genes (DEGs); and the final wave of gene activation was also involved in MCH class II and immune response (Supplementary Fig. 3, Supplementary Data 3). Collectively, by re-ordering individual cells along the developmental trajectories, Mpath analysis elucidated phase-dependent waves of transcriptional changes, and highlighted the chronological relationship of gene regulation during DC development.

### Experimental validation of Mpath results

**Figure 5:**
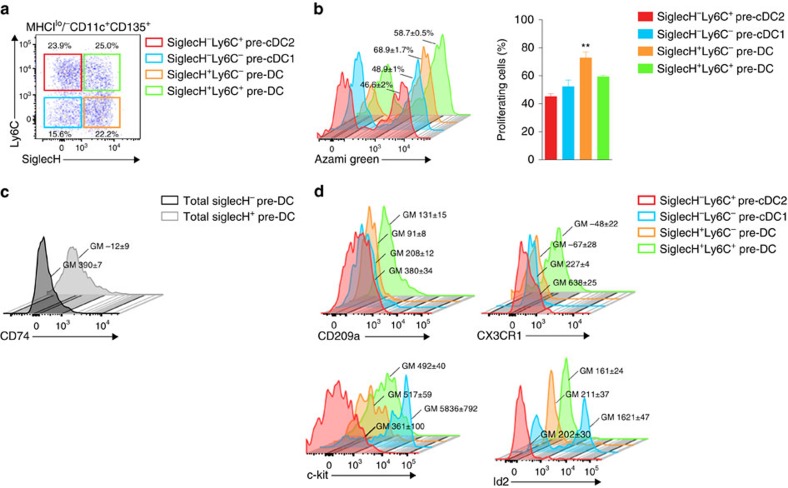
Experimental validation of Mpath results. Biological validation of preDC subpopulations predicted by Mpath. (**a**) Flow cytometry analysis of bone marrow preDC subsets: SiglecH^−^Ly6C^+^ pre-cDC2 (red), SiglecH^−^Ly6C^−^ pre-cDC1 (blue), SiglecH^+^Ly6C^−^ preDC (orange) and SiglecH^+^Ly6C^+^ preDC (green), gated on DAPI^−^Lin^−^MHCII^lo/−^CD11c^+^CD135^+^ cells. (**b**) Flow cytometry analysis (left) or frequency of proliferating cells among bone marrow preDC subsets found in **a** (right), as determined by expression of Azami Green in Fucci mice *in vivo*. The number associated with each peak indicates the percentage of proliferative (Azami Green^+^) cells **P<0.01. Flow cytometry analysis of marker gene expression as suggested by MPath of (**c**) the surface protein CD74 in total committed SiglecH^−^ (black) or uncommitted SiglecH^+^ (grey) or (**d**) the surface proteins Cd209a, Cx3cr1, c-kit and transcriptional factor Id2 on preDC populations isolated from bone marrow. The number associated with each fluorescent peak indicates the fluorescence geometric mean for the studied marker. Data are representative of two independent experiments with three replicates per condition.

Mpath analysis showed that cell cycle and proliferation genes were upregulated in early uncommitted preDCs, and downregulated in late cDC1-committed or cDC2-committed preDCs (Fig. 4, Supplementary Fig. 2b). To validate these observations, we assessed the proliferation of early uncommitted, late cDC1-committed or cDC2-committed preDCs *in vivo* in the bone marrow of Fucci (fluorescent ubiquitination–based cell-cycle indicator) reporter mice^36^. In these mice, green-emitting fluorescent protein Azami Green was fused to the protein geminin, whose expression is associated with cells in the S, G2 and M phases of the cell cycle. As reported in our previous study^9^, expression of the surface proteins SiglecH and Ly6C distinguished 4 preDC subpopulations each of which showed distinct developmental potentials. SiglecH^−^Ly6C^−^ population resembled cDC1-committed preDCs that preferentially gave rise to cDC1; SiglecH^−^Ly6C^+^ population resembled cDC2-committed preDCs that preferentially gave rise to cDC2; SiglecH^+^Ly6C^−^ and SiglecH^+^Ly6C^+^ populations resembled uncommitted preDCs that gave rise to both cDC1 and cDC2 (Fig. 5a). Therefore, we measured the frequency of proliferating cells in each preDC population in the fucci reporter model (Fig. 5b). Statistical comparison indicated that SiglecH^+^Ly6C^−^ population which marks uncommitted preDCs has significantly (adjusted *P* value<0.05, Mann–Whitney test) more proliferating cells as compared with each of the rest populations. Another uncommitted preDC population SiglecH^+^Ly6C^+^ also displayed elevated proliferation as compared with subset-committed preDCs, though the differences were not significant (adjusted *P* value>0.05, Mann–Whitney test). Our *in vivo* proliferation assay validated Mpath results that DC progenitors decreased their proliferation as they became more differentiated towards the cDC1 or cDC2 lineage. This also confirms the results achieved in our previous work^9^.

In addition, Mpath analysis identified putative new markers of cDC1- or cDC2-committed preDC subsets. We verified some of these markers by using flow cytometry. *Cd74* was identified by Mpath as a common marker for cDC1- or cDC2-committed preDCs (Fig. 3c). As indicated in our previous work^9^ and other published reports^37^,^38^, SiglecH^+^ marks a subset of preDCs that give rise to both cDC1 and cDC2, whereas SiglecH^−^ preDCs showed subset-specific priming. By flow cytometry, we gated SiglecH^+^ and SiglecH^−^ subpopulations of total preDCs, and found that the expression of *Cd74* was higher in the SiglecH^−^ population (Fig. 5c). It verifies Mpath results that *Cd74* marks late preDCs that are exclusively committed to cDC1 or cDC2. Moreover, Mpath identified *Cd209a* and *Cx3cr1* as markers of cDC2-committed preDCs (Fig. 3f). Flow cytometry analysis validated that the expression of *Cd209a* and *Cx3cr1* was the highest in SiglecH^−^Ly6C^+^ subpopulation which is cDC2-specific preDCs (Fig. 5d). On the other hand, Mpath rediscovered *Kit* and *Id2* as markers of cDC1-committed preDCs (Fig. 3e). Flow cytometry analysis verified that the expression of *Kit* and *Id2* was much higher in SiglecH^−^Ly6C^−^ subpopulation which is cDC1-specific preDCs (Fig. 5d). Interestingly, *Id2* exhibited bi-model expression in cDC1-committed preDCs, which suggests SiglecH^−^Ly6C^−^ preDCs are heterogeneous and can be further dissected into Id2^+^ and Id2^−^ subsets.

Together, by using Fucci reporter mice and flow cytometry, we were able to validate the subpopulations of preDCs predicted by Mpath. Additionally, we verified the switch from proliferation to differentiation during DC maturation, as well as several putative marker genes identified by Mpath.

### Mpath constructed the lineage of myoblast differentiation

**Figure 6:**
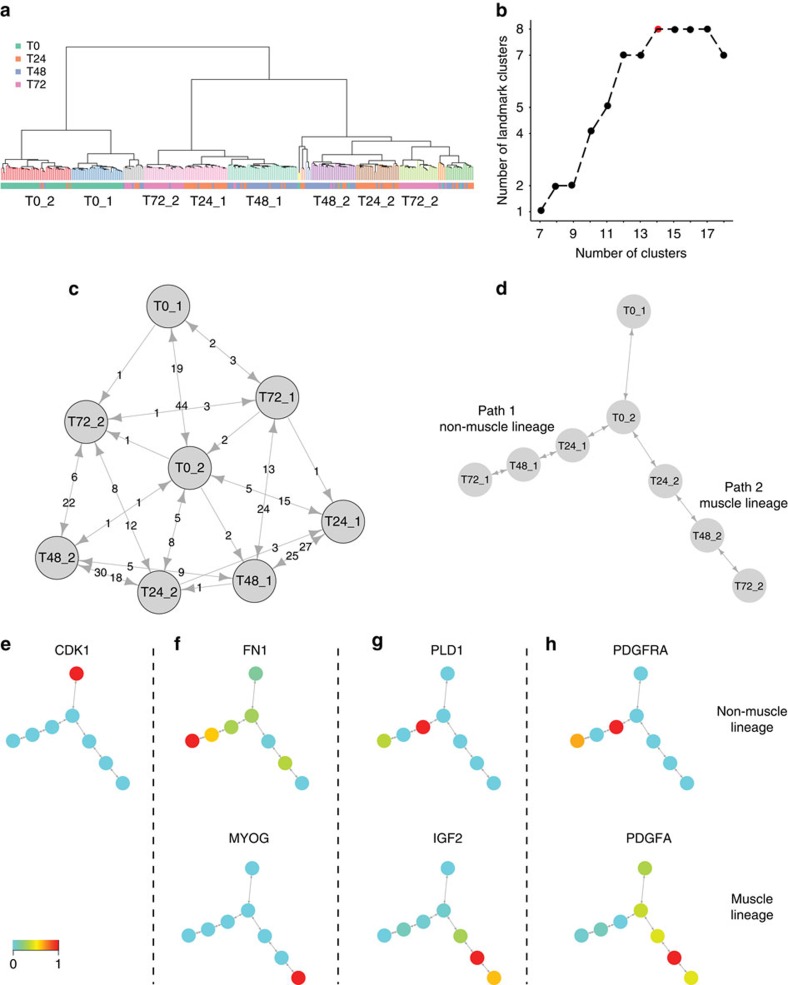
Mpath constructed multi-branching cell lineages of myoblast differentiation. Mpath was applied to single-cell RNA-sequencing data of primary human myoblasts. (**a**) Hierarchical clustering of cells and landmark designation. (**b**) Determine the optimal number of clusters by examining the plot of landmark numbers versus total number of clusters. (**c**) Weighted neighbourhood network of landmarks. (**d**) State transition network after trimming low-weighted edges. (**e**) Overlay of cell cycle marker expression on the state transition network. (**f**–**h**) Expression progression of pairs of genes for which gene–gene interactions were reported to play a role in myogenesis suggested cell–cell crosstalk between the two branches during myoblast differentiation.

We also applied Mpath to the differentiation of primary human myoblasts^10^. In this public data set we used, primary human skeletal muscle myoblasts were first expanded under high-mitogen condition and differentiation was then induced by switching to low-serum medium. Cells were captured at 0, 24, 48 and 74 h after the medium switch and analyzed using microfluidic single-cell RNA sequencing^10^. Mpath was applied to this data set using a set of DEGs from an ANOVA of these time points, having no *a priori* knowledge of the ordering of the time points. Mpath designated eight landmark states including early and late states at 0 h and two distinct states at every time point of 24, 48 and 72 h (Fig. 6a,b). It subsequently used cell counts on edges of the neighbourhood network to infer putative transitions between the landmark states (Fig. 6c,d). The resulting state transition network was able to recapitulate the process of cell differentiation from 0 to 72 h. Notably, it decomposed cell differentiation into two distinct paths starting from the late 0 h state. Markers of actively proliferating cells, such as *CDK1*, were downregulated from T0_2 onwards (Fig. 6e), indicating cell-cycle exit immediately occurred upon medium switch. Differential expression analysis revealed that cells on path 2 upregulated several well-known regulators of myogenesis, such as *MYOG* (ref. 39), *MYOD1* (ref. 39) and *MEF2C* (ref. 39), whilst cells on path 1 upregulated *PDGFRA* (ref. 40) and *SPHK1* (ref. 40; Supplementary Data 4), suggesting that only path2 differentiated into muscle cells while path 1 contained interstitial mesenchymal cells. These findings are consistent with early findings by monocle analysis^10^.

Among the DEGs between the two branches, several genes showed opposite expression trends and their interactions appear to play a role in myogenesis. An extracellular matrix molecule Fibronectin 1 (*FN1*) was found to be upregulated only at the last stage of the non-muscle path; simultaneously key marker of muscle differentiation *MYOG* was upregulated at the last stage of the muscle path (Fig. 6f). Fibronectin was recently shown to promote migration, alignment and fusion of myoblast in an *in vitro* myoblast cell model^41^. In addition, the non-muscle cells upregulated *PDL1* and mTOR signalling pathway as compared with the muscle cells, whilst extracellular molecule *IGF2* was found to be upregulated on the muscle differentiating path (Fig. 6g). *PLD1* has been reported to positively regulate mTOR signaling leading to the production of *IGF2*, an autocrine factor instrumental for the initiation of myoblast differentiation^42^. Moreover, *PDGFRα* was upregulated in the non-muscle path whilst its ligand *PDGFα* was upregulated in the muscle path (Fig. 6h). Recent reports have showed *PDGFRα* is expressed in fibrocyte precursors and increased *PDGFRα* activation can drive fibrosis^40^. Our findings strongly suggested cell–cell crosstalk between the muscle and non-muscle lineages during myoblast differentiation.

**Figure 7:**
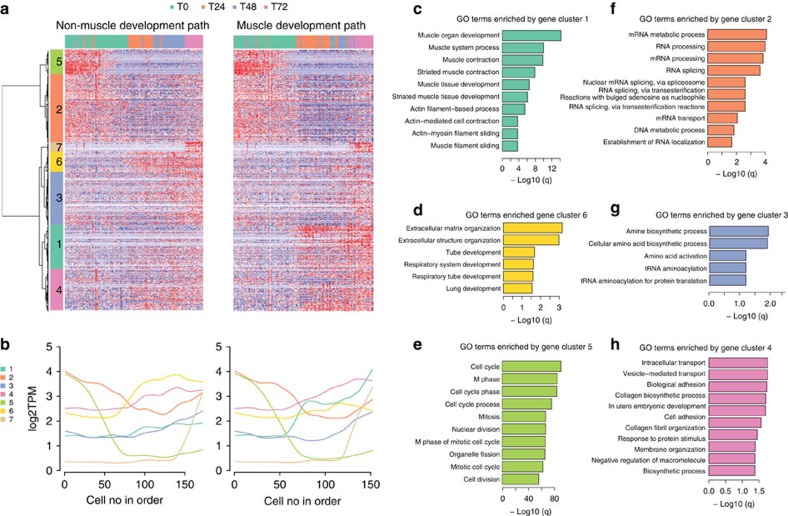
Mpath re-ordered single cells and revealed sequential waves of gene regulation during myoblast differentiation. Mpath re-ordered single cells along the muscle and non-muscle developmental trajectories respectively. (**a**) Heatmap and clustering of genes that were differentially regulated during the development from 0 to 72 h (genes in rows and cells in columns). Cells were placed in the order of increased differentiation. Clustering of genes identified seven distinct groups. (**b**) Loess smoothed line chart visualization of dynamic trends of gene expression per group with respect to pseudo-temporal re-ordering. (**c**–**h**) Gene ontology analysis identified biological processes significantly (adjusted *P* value<0.05, DAVID gene ontology analysis) enriched by genes from group 1 (**c**), group 6 (**d**), group 5 (**e**), group 2 (**f**), group 3 (**g**) and group 4 (**h**).

Mpath next re-ordered the cells along the two branches and grouped genes with similar trends in expression. This analysis revealed seven temporal waves of transcriptional changes during differentiation (Fig. 7a,b, Supplementary Data 5). Two clusters (1 and 6) of genes showed distinct expression kinetics along the muscle versus non-muscle development paths (Fig. 7a,b). Gene cluster 1, enriched for muscle development process, was upregulated only on the muscle differentiation path (Fig. 7a–c). Gene cluster 6 was upregulated only on the non-muscle path, and was enriched for extracellular matrix organization and included genes encoding collagen (Fig. 7a,b,d). Other than clusters 1 and 6, the remaining clusters followed almost identical dynamic trends on both branches. Gene cluster 5 comprising mostly cell cycle genes was rapidly downregulated immediately after the earliest state T0_1 (Fig. 7a,b,e). Genes from cluster 2 were gradually downregulated and were largely involved in RNA processing and splicing (Fig. 7a,b,f). Genes from cluster 3 were gradually upregulated involving in amino acid synthesis and protein translation (Fig. 7a,b,g). Genes from cluster 4 were also gradually upregulated involving in intracellular transport and cell adhesion (Fig. 7a,b,h). Such synchronized gene expression patterns again implied close cell–cell interaction and coordination between the two branches.

### Comparing Mpath with existing methods

We compared Mpath with SCUBA, monocle, diffusion map, PCA and MST on the DC progenitor data set, and evaluated their performance based on published experimental data^9^. Our comparison showed that Mpath produced more biologically relevant results and it is the only method that faithfully recapitulates the branching of cDC1- and cDC2-committed preDCs.

**Figure 8:**
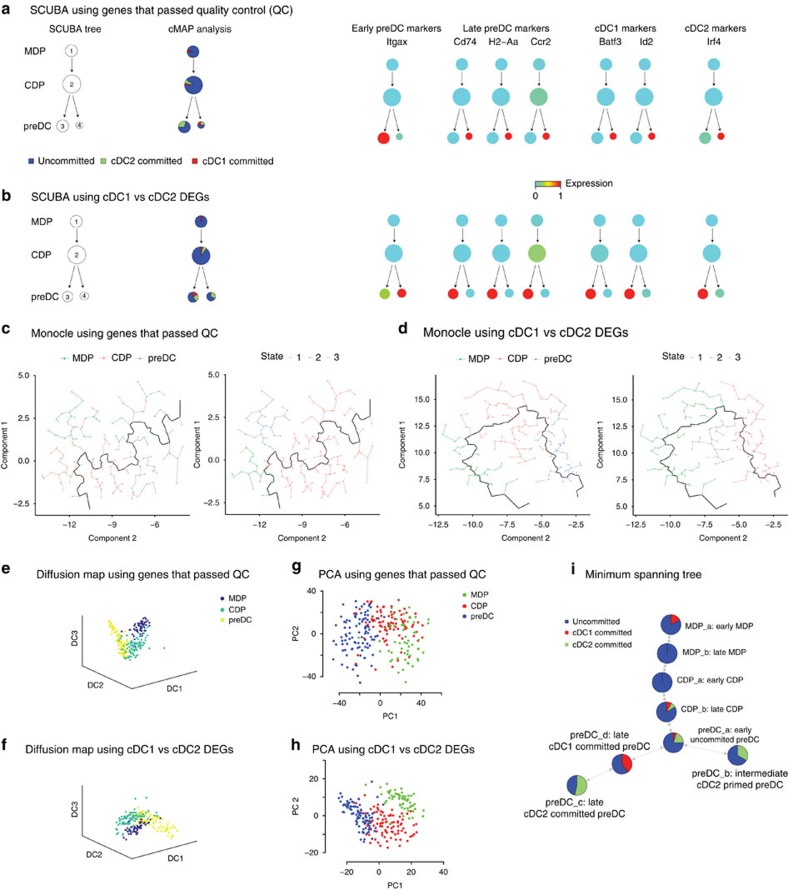
Comparing Mpath with existing methods. SCUBA, monocle, diffusion map, PCA and MST were applied to our single-cell RNA-sequencing data set of DC progenitors. (**a**–**b**) Bifurcation trees generated by SCUBA using all genes that passed quality control (QC) and DEGs between mature cDC1 and cDC2 respectively; the pie's represent proportion of cells that were uncommitted, cDC1-committed or cDC2-committed as identified via cMAP analysis; the panel on the right shows overlay of early preDC, late preDC, cDC1 and cDC2 marker expression on the SCUBA trees. (**c**,**d**) Monocle analysis using all genes that passed QC and DEGs between mature cDC1 and cDC2 respectively. (**e**–**f**) Diffusion map analysis using all genes that passed QC and DEGs between mature cDC1 and cDC2 respectively. (**g**,**h**) PCA using all genes that passed QC and DEGs between mature cDC1 and cDC2 respectively. (**i**) MST-derived state transition network using Euclidean distances between the averages of cells in each landmark cluster.

*Comparing Mpath with SCUBA*. We applied SCUBA (ref. 15) to the single-cell RNA-seq data of DC progenitors using genes that passed quality control (Fig. 8a) and mature cDC1 versus cDC2 DEGs (Fig. 8b). In both cases, SCUBA identified MDP cluster 1, CDP cluster 2, preDC clusters 3 and 4. And it derived a binary tree structure in which a bifurcation event stems from CDP cluster 2 giving rise to preDC clusters 3 and 4 on separate branches. SCUBA preDC cluster 3 mainly consisted of cells from Mpath defined early and intermediate preDCs; cluster 4 mainly consisted of cells from Mpath defined late preDCs that were committed to cDC1 or cDC2 (Supplementary Fig. 4a). To better annotate SCUBA preDC clusters 3 and 4 for their biological relevance, we performed a thorough examination of cells in these two clusters using several approaches. Firstly, we performed cMAP analysis using DEGs between mature cDC1 versus cDC2 and calculated the proportion of cDC1- or cDC2-committed cells in each cluster. cMAP analysis showed that preDCs on one branch contain both cDC1 and cDC2-committed cells, while preDCs on the other branch contain cDC2-committed cells. Secondly, we overlaid median expression of known markers on SCUBA tree. In the case of using genes that passed QC (Fig. 8a), early preDC marker *Itgax* was upregulated only in preDC cluster 3, while genes including *Cd74*, *H2-Aa* and *Ccr2* which are known to mark late preDCs were upregulated only preDC cluster 4. The progression of these DC maturation markers suggests preDC cluster 3 is early preDCs while preDC cluster 4 is late preDCs. Furthermore, the well-known cDC1 markers *Batf3*, *Id2* and cDC2 marker *Irf4* were upregulated only in preDC cluster 4, suggesting preDC cluster 4 are subset committed while cluster 3 are uncommitted. Lastly, differential expression analysis between SCUBA preDC clusters 3 and 4 revealed that genes upregulated in cluster 3 were similar to an uncommitted preDC signature^9^ (Supplementary Data 6), whereas genes upregulated in cluster 4 included both cDC1 and cDC2 markers^9^ (Supplementary Data 7). These observations strongly indicate the two branches identified by SCUBA represented early uncommitted and late committed preDCs, but not cDC1- or cDC2-committed preDC subsets. Similar results were observed when SCUBA was applied using mature cDC1 versus cDC2 DEGs (Fig. 8b). What SCUBA found is not in agreement with previous report^9^ wherein solid experimental validation showed preDC subsets made exclusive pre-commitment to cDC1 or cDC2. In contrast, Mpath-derived state transition network was able to recapitulate the branching event that starts from early preDC and arrives at cDC1- or cDC2-committed preDCs separately.

*Comparing Mpath with monocle*. We applied monocle to the same DC progenitor data set using genes that passed quality control (Fig. 8c) and mature cDC1 versus cDC2 DEGs (Fig. 8d). When applied with genes that passed QC, the backbone of monocle-derived tree connected mainly CDP and preDC cells, and majority of MDP cells were placed on branches (Fig. 8c). When informed to construct paths with two end states, monocle identified one end state being preDC on the backbone and the other end state being MDP on a side branch. This is inconsistent with the well-known DC development cascade from MDP to CDP and then preDC. When applied with mature cDC1 versus cDC2 DEGs, monocle identified a backbone path that connects majorities of MDPs, CDPs and preDCs in cascade (Fig. 8d). When informed of two end states, monocle identified a divergent branch that comprised a subpopulation of CDPs. Still, monocle was unable to uncover the previously reported branching event of preDC commitment to cDC1 or cDC2 (ref. 9).

*Comparing Mpath with diffusion map and PCA*. We applied diffusion map^14^ and PCA to the same DC progenitor data set using genes that passed quality control (Fig. 8e,g) and mature cDC1 versus cDC2 DEGs (Fig. 8f,h). Both methods were able to visualize the developmental continuum spanning from MDP through CDP to preDC. However, no evident branching structure was detected even when DEGs between mature cDC1 and cDC2 were used. These two methods are not sensitive enough to uncover cDC1- or cDC2-committed preDC subsets.

*Comparing Mpath with MST approach*. When applied to the DC progenitor data set, Mpath identified two subsets of late preDCs, that is, preDC_c and preDC_d, which were exclusively committed to cDC1 or cDC2. Whilst they belonged to two separate lineages, both are late differentiated preDCs and hence shared high transcriptional similarities in term of cellular maturation. This can also be seen in the hierarchical clustering, in which landmarks preDC_c and preDC_d were grouped together (Fig. 2a). Hierarchical clustering is usually unable to inform the developmental relatedness between clusters. An alternative approach called MST has been adopted by several methods such as SPADE (ref. 43) and monocle^10^ to infer developmental relatedness between cell subsets or individual cells. MST approach utilizes point-to-point distances and identifies the shortest path to connect all the cells or cell subsets. Here we applied the conventional MST approach based on pair-wise Euclidean distances between landmarks to infer the developmental relatedness between landmark clusters. The resulting network placed landmarks preDC_c and preDC_d (that is, cDC1- or cDC2-committed preDCs) on the same branch whilst landmark preDC_b (that is, intermediate cDC2 primed preDCs) on the opposite branch (Fig. 8i). This implies cDC1-committed preDCs give rise to cDC2-committed preDCs, which is unlikely to be true. It has been reported solid experimental data showing that subset committed preDCs exclusively give rise to only cDC1 or cDC2 (ref. 9). These discrepancies mark the limitation of conventional distance-based MST approaches. While in Mpath, rather than point-to-point distances, we use the count of cells on transitional stage as an indicator of proximity between landmark states. This strategy of Mpath heuristically reflects the continuum nature of cellular development and the fact that cells progress between states along a continuous path. Hence, by using transitioning cells to pave the pathways from early progenitors, Mpath was able to split the two transcriptionally similar end stages preDC_c and preDC_d into separate branches, which has been biologically proven. Our comparison highlights the advantages of Mpath over MST approach in delivering relevant biological insights.

In summary, our comparison shows that the existing methods either failed to produce biologically relevant results or were only able to map the progression from MDP to CDP to preDC. Their common limitation is that they are unable to detect the branching event wherein subsets of preDCs are exclusively committed to cDC1 or cDC2 lineage, which has been reported and supported with solid experimental data^9^. The reason is mainly due to the fact that the differences between cDC1 and cDC2-committed preDCs are subtle, and the existing methods lack the sensitivity to uncover these subtle but vital differences. In contrast, our Mpath method by applying the concept of neighbouring cells on transitional stages is able to rebuild the developmental paths from MDP to CDP and then early uncommitted preDC towards cDC1- or cDC2-committed preDC. This highlights the advantage of our neighboring-state-transition based concept over existing dissimilarity- or distance-based approaches.

### Robustness of Mpath

**Figure 9:**
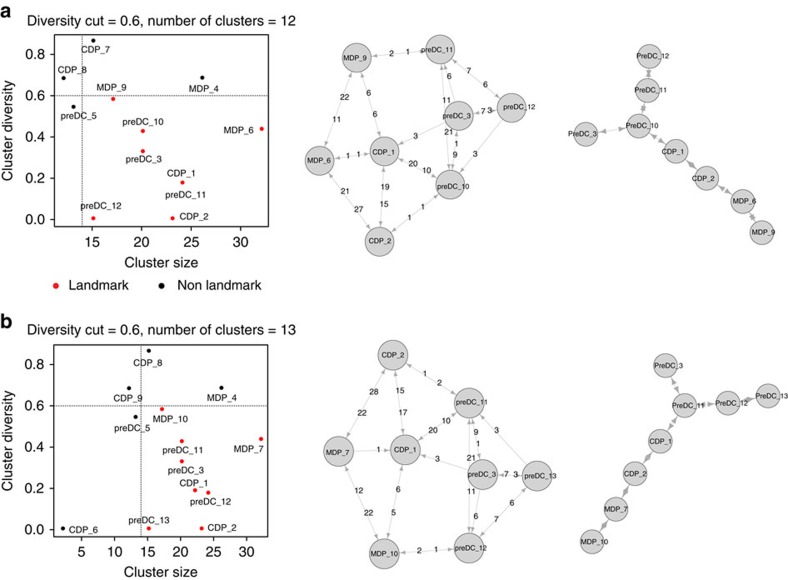
Mpath constructed reproducible cell lineages when the analysis was repeated with different initial numbers of clusters. We evaluated the robustness of Mpath by repeating the analysis of the DC progenitor data set using different initial numbers of clusters. (**a**) Mpath results when initial number of cluster is 12. (**b**) Mpath results when initial number of cluster is 13.

The number of clusters initially considered by Mpath is determined by the ‘cut' position in the hierarchical clustering dendrogram. We first assessed Mpath's robustness over different number of clusters by cutting the dendrogram iteratively with increasing depth. The number of landmark clusters distinguished from the entire cluster set first increased linearly with the number of clusters, subsequently plateaued when the number of clusters was 11, 12 or 13 (Fig. 2b), but then decreased when the number of clusters was greater than 13. When we repeated the analysis by cutting the dendrogram at different levels to generate 12 or 13 clusters, Mpath was able to designate the same set of landmarks and derive reproducible state transition networks (Fig. 9).

**Figure 10:**
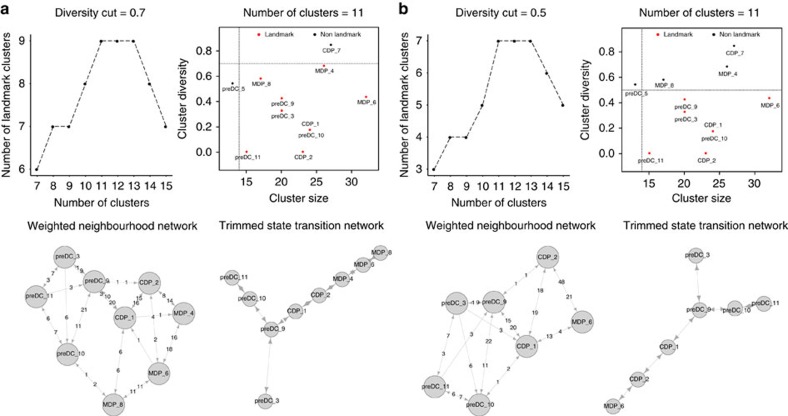
Mpath constructed reproducible cell lineages when the analysis was repeated with different diversity cut for distinguishing landmark clusters from non-landmark clusters. We evaluated the robustness of Mpath by repeating the analysis of the DC progenitor data set using different diversity cut for distinguishing landmark clusters. (**a**) Mpath results when diversity cut is 0.7. (**b**) Mpath results when diversity cut is 0.5.

We continued to assess Mpath's robustness over different diversity cuts for distinguishing landmark clusters from the entire cluster set (Fig. 10). When we increased the diversity cut from 0.6 to 0.7, the landmark number versus total number of clusters plot showed the same pattern as when diversity cut 0.6 was applied. The number of landmark clusters also first increased and then plateaued when the number of clusters was 11, 12 or 13 and decreased afterwards (Fig. 10a). Due to a less stringent diversity cut 0.7, the maximum number of landmark clusters increased to nine. Most importantly, Mpath reproduced similar landmark cluster set and similar state transition network, which was still able to recapitulate the development cascade from MDP through CDP to preDC as well as the branching event from early preDC to cDC1- or cDC2-committed preDC. The only difference is that a new landmark MDP_4 was added between MDP_6 and CDP_2, offering a higher-resolution delineation of MDP heterogeneity and development. When we decreased the diversity cut to 0.5, the landmark number versus total cluster number plot reproduced the same pattern as previous (Fig. 10b). The optimal number of clusters was still 11, but the number of landmark clusters reduced to seven. Nevertheless, with 7 landmarks, Mpath was still able to reconstruct the correct state transition network (Fig. 10b). The only change is losing one MDP landmark state, which does not affect the main structure of DC progenitor development pathway.

Collectively, Mpath was found to be robust and to generate similar networks when input parameters such as diversity cut and initial cluster number were changed.

## Discussion

The Mpath algorithm is able to resolve multi-branching single-cell developmental trajectories, allowing for multiple cell fates stemming from a single progenitor cell type. Applied to mouse bone marrow-derived DC precursors, Mpath reconstructed a multi-branching state transition network of DC development and differentiation. Our study revealed early subset-commitment emerged from late CDP stage onwards and a branching event occurred at the preDC stage. The branching event led to two preDC subsets that were exclusively committed to cDC1 or cDC2 lineages. In addition, Mpath identified early transcriptional markers for cDC1 and cDC2 subset commitment respectively. These markers comprised known subset-specific genes and putative novel regulators that were unknown to act in differentiation. Lastly, it placed individual cells in order along the developmental trajectories, revealing a switch from proliferation to differentiation during DC maturation. Furthermore, its ordering of single cells discovered phase-dependent waves of gene regulation on the scale of DC development and subset differentiation continuum.

During multi-lineage differentiation, individual cells make divergent fate decision responding to various environmental cues or unequally partitioned signalling molecules during asymmetric cell division^4^. As demonstrated by the analysis of DC progenitor cells, Mpath is able to predict individual cell's fate using signature genes of differentiated cell types. The algorithm travels back in time from differentiated cell subsets and maps the developmental path from known terminal differentiated cell subsets back to its progenitors. By doing so, Mpath identifies subset-committed progenitor cells and early key regulators of subset differentiation. Progenitor cells are usually rare and hence bulk transcriptomic analysis is often not applicable. Mpath together with single-cell RNA sequencing enables us to dissect early cellular development and lineage commitment at progenitor stages.

As recent studies are carried out at single-cell resolution, it has been discovered cellular continuums which span across different cell populations^9^,^10^. Cellular development is now increasingly recognized as a continuous process characterized by transitional stages. For two adjacent stages along the developmental lineage, one should expect neighbouring cells that are developing from one stage towards the next. Inspired by this continuum concept, Mpath uses count of neighbouring cells to estimate potential state transition. Via neighborhood-based cell state transition mapping, Mpath is able to resolve complex nonlinear developmental lineages that could not be explained by pair-wise transcriptional dissimilarities. Notably, it provides sufficient sensitivity to reveal subtle but relevant differences in addition to mapping more dominant changes of global progression. In the case of our DC progenitor study, the differences between cDC1 and cDC2-committed preDCs were critical but subtle compared with the differences between early and late preDCs. Though existing algorithms were able to recapitulate the global progression from MDP to CDP to early preDC and lastly late preDCs, they could not uncover the subtle divergence of subset commitment owing to the fact that the differences between early and late preDCs override the differences between cDC1- and cDC2-committed preDCs. In contrast, Mpath was able to delineate the two separate branches of cDC1 and cDC2 subset commitment respectively and meanwhile map the more dominant progression from early to late preDCs.

Collectively Mpath provides a general framework for detecting nonlinear multi-branching developmental trajectories from single-cell RNA-sequencing data without requiring *a priori* knowledge of the temporal ordering of developmental subsets, and utilizing genes only from end-stage contrasts. Our study has demonstrated its utility in data sets of mouse bone marrow-derived DC progenitors and primary human myoblasts. In future, we will extend the Mpath algorithm for development processes starting from multiple independent progenitor cell types, for more heterogeneous single-cell data sets in which specific subset information is not known.

## Methods

### Mpath constructs multi-branching cell lineages

We implemented the Mpath methodology into an R package and will submit it to bioconductor. The Mpath R-package and all data used for this study are publicly available for download at https://github.com/JinmiaoChenLab/Mpath. A reference manual of Mpath is given in Supplementary Software. As illustrated in Supplementary Fig. 1, Mpath takes as inputs TPM, RPKM or FPKM values that are generated by third party programs, and constructs cell lineages using the following steps.

### Landmark designation

Mpath first performs unsupervised hierarchical clustering of cells using Euclidean distance and Ward.D agglomeration method. It subsequently generates cluster sets by iterative cutting of the dendrogram at increasing depth. Each cluster set is then ranked according to ‘purity', using the *a priori* FACS sorting information and Shannon's index, and size (relative number of cells in the cluster). A cluster is then designated as a landmark cluster if it passes the empirically determined cutoffs of 0.6 and 5% for purity and size, respectively. Examination of a plot of landmark numbers versus total number of clusters for each cluster set (Figs 2b and 6b) revealed an inflection point at which landmark numbers reached a plateau and then decreased despite increasing numbers of clusters. Mpath uses this information to determine the optimal cut of the dendrogram. An average per gene expression for each of the designated landmark clusters was then generated from all of the single cells in the respective clusters. These averages which represent canonical cellular states were referred to as ‘landmarks'. The landmarks were named by the major cell type in the cluster.

Mpath defines landmarks as cell clusters that are of high purity and reasonable size. More landmarks are favourable as it increases the resolution of cellular states. With more clusters delineated, cluster purity increases while size decreases. The number of landmarks first increases and then reaches a stabilized phase. Mpath achieves the optimal number of landmarks at the point where the number of landmarks starts to plateau whilst number of clusters increases. By using high purity and large size as criteria for landmark designation, Mpath overcomes the key challenge for any clustering algorithm, which is defining the optimal number of clusters without a prior knowledge of the underlying cell subsets.

### Construct weighted neighbourhood network of landmarks

Theoretically, cells that are transitioning from one to another state share similarity in gene expression with both end states^9^,^10^,^11^. Mpath thus calculates the Euclidean distance of each cell to the landmarks. Subsequently each cell is placed in between its two nearest neighbouring landmarks. Based on the count of cells placed between each pair of landmarks, Mpath constructs a weighted neighbourhood network in which nodes represent landmark states; edges connecting landmarks are weighted by the number of neighbouring cells. Rather than pair-wise correlation or distance, Mpath uses the number of neighbouring cells to estimate the likelihood of there existing a true transition between states.

### Trim the neighbourhood network to state transition network

Single-cell RNA-seq data is noisy as a result of technical and biological variability^8^,^44^,^45^, including low amount of starting material; amplification bias; and cell-to-cell variation in sequencing efficiency. In addition, inherently stochastic gene expression can cause heterogeneity among otherwise-identical cell populations. In the neighbourhood network derived by Mpath, some connections made between landmarks can be artifacts due this noise. To account for this, every connection in the network is weighted by *w*(*e*), the number of cells that support the state transition. A higher number of cells increase the likelihood of the reality of putative transition between the landmark states. The Mpath algorithm trims the neighbourhood network to remove connections that have low cell count support. Rather than choosing an arbitrary cutoff value of cell numbers required for a transition to be accepted, Mpath uses a heuristic approach to achieve an optimal trimmed network that connects all landmarks and includes the maximum number of transitional cells by using minimal number of edges. The trimming process uses −*w*(*e*) to measure the distance between landmarks, and applies MST to find the shortest path that connects all the landmarks. The resulting trimmed network is used to present a candidate state transition network of cellular development.

### Landmark annotation

To annotate the trimmed state transition network with respect to the developmental system being studied, Mpath identifies DEGs between landmark cells via ANOVA analysis. We adjusted the *P* values for multi-test correction using Benjamini–Hochberg and identified DEGs using adjusted *P* values threshold 0.05 (Supplementary Data 1, Supplementary Data 8). We then performed gene ontology analysis of DEGs using DAVID: Functional Annotation Tools; and identified significantly enriched biological processes using Benjamini adjusted *P* value threshold 0.05. Based on gene ontology enrichment analysis of DEGs, we inferred the biological processes involved at different cellular states and annotated the landmarks as biologically meaningful cell populations. Furthermore, Mpath overlays the median expression of marker genes on the state transition network to visualize and verify the progression of gene expression along the developmental paths.

In addition to differential expression analysis, Mpath annotates the landmarks via cMAP (ref. 18) analysis using signature genes derived from literature or bulk transcriptomics data. Signature genes for mature cDC2 and cDC1 were identified from microarray data set GSE60783. They were used respectively as up and downregulated genes to perform cMAP analysis to each single-cell. The *P* values were calculated through 1,000 permutations. Cells whose gene expression profile was significantly correlated with signature genes were selected by *P* value<0.05. cMAP scores were scaled to the range from −1 to 1. Cells with positive cMAP score are correlated with cDC2; cells with negative cMAP scores are correlated with cDC1. By cMAP analysis, Mpath identifies cDC1 or cDC2 pre-committed cells at MDP, CDP and preDC stages.

### Re-ordering of single cells

To generate a more fine grained analysis, and move from clusters back to the single-cell information, Mpath re-orders individual cells along the developmental trajectories. Mpath computationally reconstructs cell developmental pathways as a multi-destination journey on a map of connected landmarks wherein individual cells are placed in order along the paths connecting the landmarks (Supplementary Fig. 5a). Given a sequence of landmarks (*a*, *b*, *c*, *d*) along a development path, Mpath places the cells in order on the edge connecting the adjacent landmarks. It first identifies cells (*ab*_1_, *ab*_2_,…, *ab*_*i*_,…) that are potentially transitioning from landmark *a* to *b* based on their transcriptional proximities to both landmarks. To determine the ordering of these cells along the transition from landmark *a* to *b*, Mpath locates their projection points on the line spanning landmark *a* and *b* (Supplementary Fig. 5b). A given cell *ab*_1_ is orthogonally projected to the line spanned by landmarks *a* and *b*, and the projection point *x*_1_ is identified. Mpath repeats this process for individual cells (*ab*_1_, *ab*_2_,…, *ab*_*i*_,…) and identifies their respective project points (*x*_1_, *x*_2_,…, *x*_*i*_,…). Cells (*ab*_1_, *ab*_2_,…, *ab*_*i*_,…) are then sorted according to the ordering of (*x*_1_, *x*_2_,…, *x*_*i*_,…) with respect to landmarks *a* and *b*. The above procedure is then repeated for every two adjacent landmarks, that is, (*b*, *c*) and (*c*, *d*), giving rise to a concatenated pseudo-temporal ordering (*ab*_1_, *ab*_2_,…, *ab*_*i*_,…) (*bc*_1_, *bc*_2_,…, *bc*_*i*_,…) (*cd*_1_, *cd*_2_,…, *cd*_*i*_,…) along the developmental path spanning landmarks *a*, *b*, *c* and *d*. The same process is repeated for the developmental path spanning landmarks *a*, *b*, *c* and *e*. If a cell is ‘before' the first landmark or ‘after' the last landmark, we placed it on the extension of the edges connecting its two nearest landmarks (Supplementary Fig. 5c).

### Single-cell RNA-sequencing data mapping and preprocessing

Short reads were aligned to mouse reference genome MM10 using RSEM (ref. 46). Transcripts per million reads (TPM) values were calculated by RSEM using gencode annotation version M4. TPM values were transformed to log2 scale. Outlier cells were identified by SINGuLAR toolsets and were excluded for downstream analysis. Low expression genes that have TPM values <1 in more than 95% of cells in each group were excluded.

### Differential expression analysis

For the DC progenitor data set, we applied Mpath using mature cDC1 versus cDC2 DEGs derived from microarray data of splenic DCs. Microarray data were quantile normalized and DEGs were identified using Limma^47^. The DEGs include 1920 cDC2 upregulated and 2,180 downregulated genes (contrasted with cDC1). For the human myoblasts data set, we performed ANOVA analysis of cells at 0, 24, 48 and 72 h and used the ANOVA DEGs for Mpath analysis. We have used adjusted *P* value threshold 0.05 for DEG selection and the *P* values were adjusted for multi-test correction using Benjamini–Hochberg method. Analyses were performed in R version 3.0.2/Bioconductor.

### Preparation of cell suspension and flow cytometry

Bone marrow was flushed from the femur and tibia of one leg and was used after red blood cell lysis using eBioscience RBC lysis buffer. Multi-parameter analyses of labeled cell suspensions were performed on an LSR II (Becton Dickinson) and data were analyzed with FlowJo software (TreeStar). Fluorochrome- or biotin-conjugated monoclonal antibodies (mAbs) to the following were used: mouse IA/IE (M5/114.15.2) and CD172α (P84) (both from BD Biosciences); CD11c (N418), CD209a (MMD3), CD117 (2B8), Ly6C (HK1.4), SiglecH (440c), B220 (RA3-6B2), CD135 (A2F10), CD49b (DX5) and CD19 (eBio1D3) (all from eBioscience); Ly6G (1A8) and CD3e (145-2C11); CD74 (OX6) (from Abcam); and CX3CR1 (Catalog # FAB5825P) (from RnD Systems). The streptavidin–phycoerythrin-CF594 conjugate (25-4317-82) was from BD Biosciences. The dilution of antibodies was as follows: CD117 (2B8) 1:100, CD135 (A2F10) 1:100, CD74 (OX6) 1:50, CX3CR1 (Catalog # FAB5825P) 1:50, streptavidin–phycoerythrin-CF594 conjugate (25-4317-82) 1:400, all other antibodies 1:200.

### Data availability

Data utilized in this study are available in Gene Expression Omnibus with the accession codes GSE60781 (single-cell RNA-sequencing data of mouse DC progenitor cells), GSE52529 (single-cell RNA-sequencing data of human myoblast cells), GSE60782 (microarray data of mouse cDC1 and cDC2 cells). The Mpath R-package and all data used for this study are publicly available for download at https://github.com/JinmiaoChenLab/Mpath.

## Additional information

**How to cite this article:** Chen, J. *et al.* Mpath maps multi-branching single-cell trajectories revealing progenitor cell progression during development. *Nat. Commun.* 7:11988 doi: 10.1038/ncomms11988 (2016).

## Supplementary Material

Supplementary Information Supplementary Figures 1-5 and Supplementary Table 1

Supplementary Data 1 Supplementary Data 1 include differentially expressed genes (DEGs) between Mpath designated landmark preDC_a (early uncommitted preDCs), preDC_c (late cDC2- committed preDCs, i.e. pre-cDC2) and preDC_d (late cDC1-committed preDCs, i.e. pre-cDC1). The DEGs were identified using ANOVA followed by post-hoc Tukey's test. P values were adjusted for multi-test correction using Benjamini-Hochberg. Genes that were differentially expressed between any two landmarks were included. Cluster analysis of the DEGS identified 5 distinct clusters as shown in Supplementary Figure 2a. The gene cluster column indicates which cluster individual genes belong to (Supplementary Figure 2a). The marker type column labels individual DEGs as markers for early pre-DC, pre-cDC1, pre-cDC2, or pre-cDC1 and precDC2 in common. The Location and Type columns indicate the location and type of the protein- product of each gene as defined by Ingenuity Pathway Analysis (IPA). The rest columns indicate the log2 fold change and adjust p values for the comparisons between each pair of landmarks.

Supplementary Data 2 Supplementary Data 2 include genes that were differentially expressed along the development from early CDP to late cDC2-committed preDC. Clustering of these genes identified 7 distinct clusters as shown in Figure 4a. The cluster column indicates which clusters individual genes belong to.

Supplementary Data 3 Supplementary Data 3 include genes that were differentially expressed along the development from early CDP to late cDC1-committed preDC. Clustering of these genes identified 6 distinct clusters as shown in Supplementary Figure 3. The cluster column indicates which clusters individual genes belong to.

Supplementary Data 4 Supplementary Data 4 include genes that were differentially expressed between cells on the muscle differentiating path versus cells on the non-muscle differentiation path. The "non_muscle vs muscle Log2 FC" column indicates the non_muscle vs muscle fold change on log2 scale.

Supplementary Data 5 Supplementary Data 5 include genes that were either differentially expressed along the muscle differentiating path or the non-muscle differentiating path. Clustering of these genes identified 7 distinct clusters as shown in Figure 7a. The cluster column indicates which clusters individual genes belong to.

Supplementary Data 6 Supplementary Data 6 include genes that were down-regulated in SCUBA cluster 4 as compared to SCUBA cluster 3. The "Cluster 4 vs Cluster 3 Log2FC" column indicates Cluster 4 vs Cluster 3 fold change in log2 scale.

Supplementary Data 7 Supplementary Data 7 include genes that were up-regulated in SCUBA cluster 4 as compared to SCUBA cluster 3. The "Cluster 4 vs Cluster 3 Log2 FC" column indicates Cluster 4 vs Cluster 3 fold change in log2 scale.

Supplementary Data 8 Supplementary Data 8 include genes that were differentially expressed between Mpath landmark preDC_c and preDC_b, and Genes that were differentially expressed between Mpath landmark preDC_a and preDC_b. preDC_a represents early uncommitted preDC, preDC_b represents intermediate cDC2 primed preDC, preDC_c represents late cDC2 committed preDC.

Supplementary Software Mpath reference manual provides detailed documentation of Mpath R package. It covers how to install Mpath package, how to call Mpath functions and what are the expected outputs. It also includes example codes for performing Mpath analysis on the two datasets that were used in our manuscript.
