# Supplementary material for: Mpath maps multi-branching single-cell trajectories revealing progenitor cell progression during development
Source: Nat Commun. 2016 Jun 30;7:11988. doi: 10.1038/ncomms11988 (PMC4931327; doi:10.1038/ncomms11988)
Supplement: Supplementary Information — Supplementary Figures 1-5 and Supplementary Table 1 [file ncomms11988-s1.pdf]

Supplementary information

Supplementary Figures

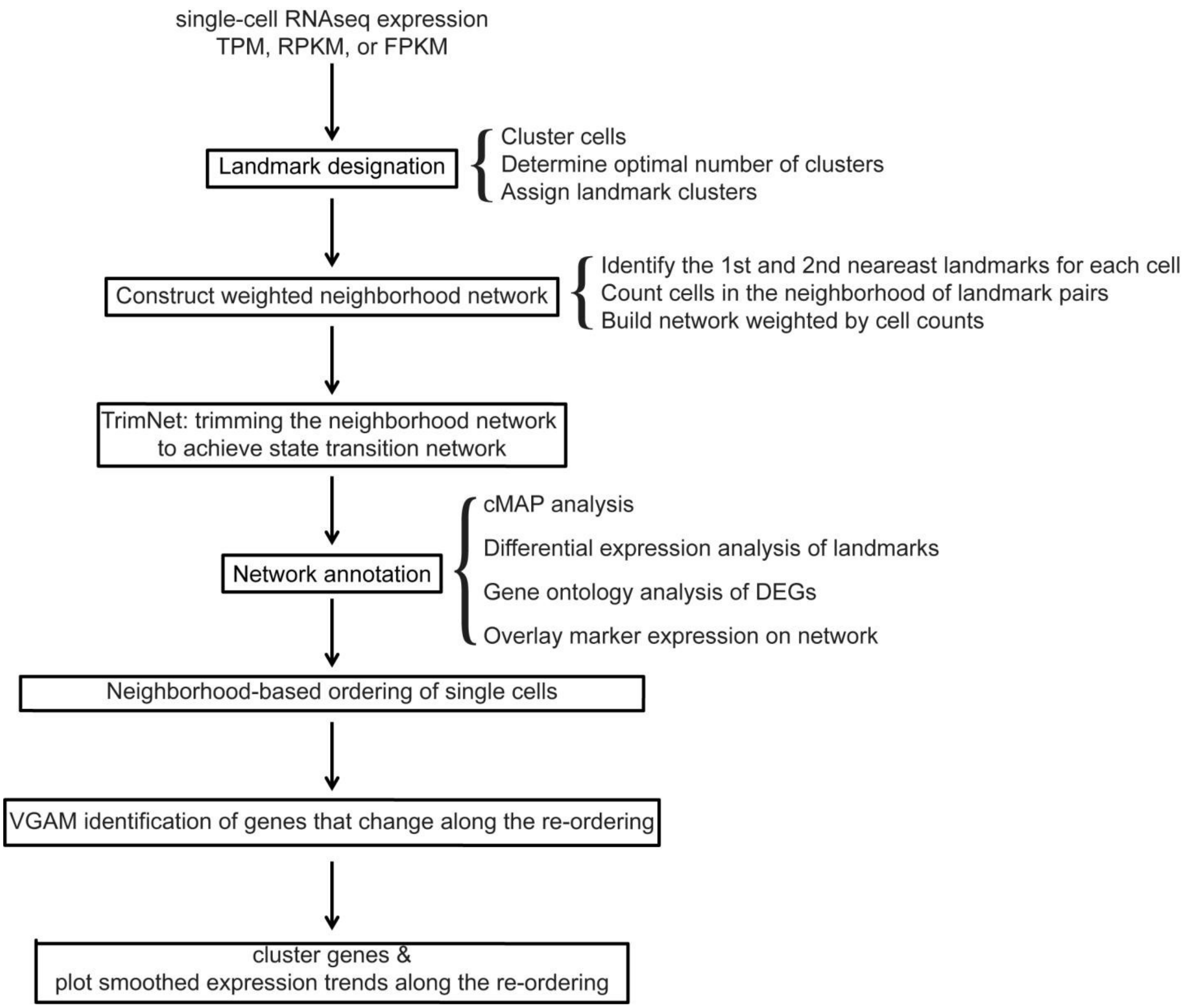

Supplementary Figure 1. Mpath: an algorithm for constructing multi-branching cell lineages from single-cell data.

Shown is a schematic diagram of Mpath, which comprises i) landmark designation, ii) construction of weighted neighborhood network, iii) TrimNet, iv) network annotation, v) neighborhood-based reordering of single cells, vi) VGAM identification of genes that change along the re-ordering, vii) clustering of genes and plot smoothed expression profiles.

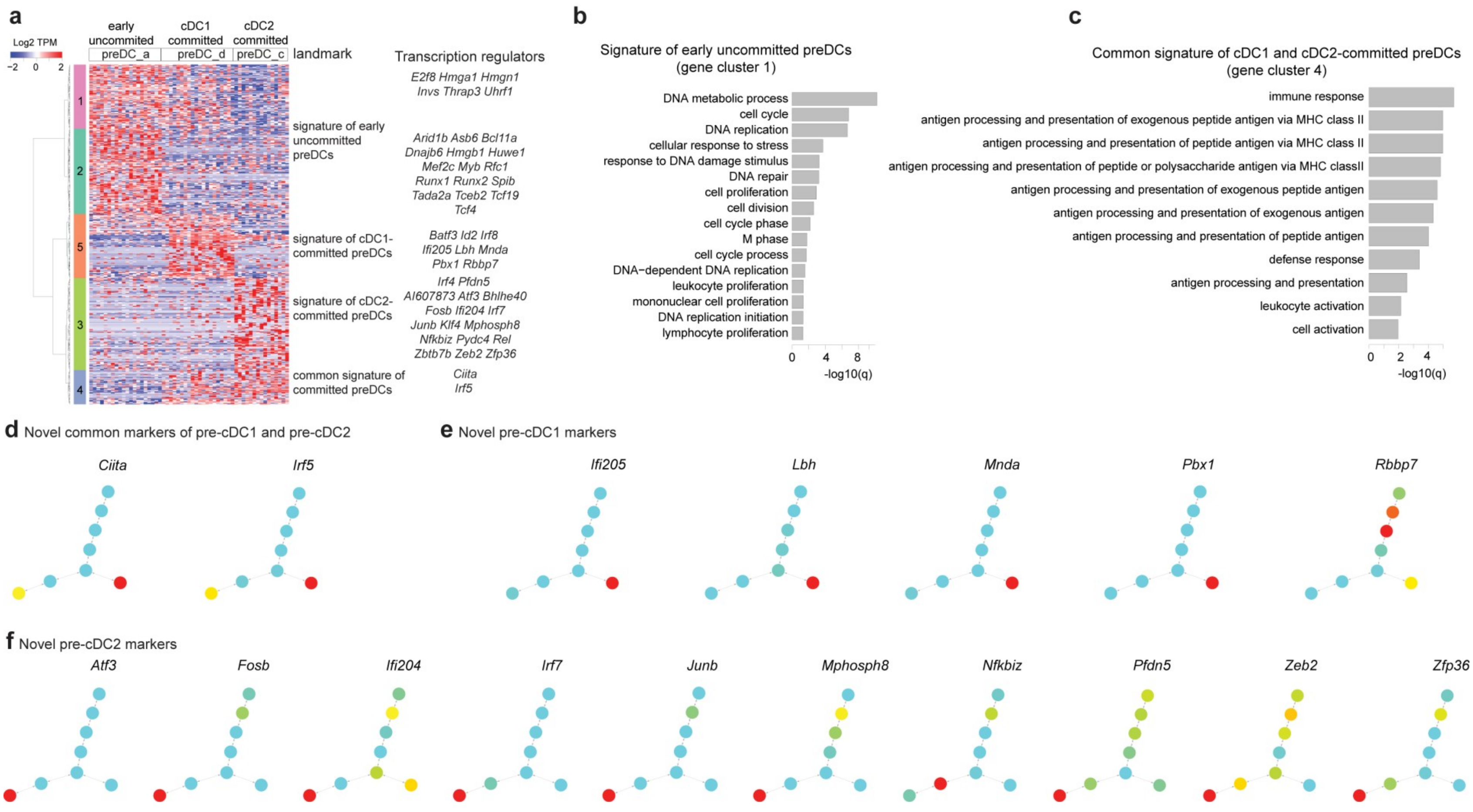

Supplementary Figure 2. Mpath identified signature genes of preDC subsets, and performed gene ontology (GO) analysis.

Mpath identified differentially expressed genes (DEGs) between landmark preDC_a, preDC_c and preDC_d using ANOVA (a) Heatmap and hierarchical clustering of DEGs. DEGs were grouped into 5 distinct clusters. Signature genes of preDC_a comprised genes from cluster 1 and 2. Genes from cluster 4 constituted common signatures of preDC_c and preDC_d. Genes from cluster 3 and 5 were exclusive signatures of preDC_c and preDC_d respectively. (b) GO analysis identified biological processes enriched by genes from cluster 1. (c) GO analysis identified biological processes enriched by genes from cluster 4. (d) – (f) Overlay of novel marker expression on the state transition network.

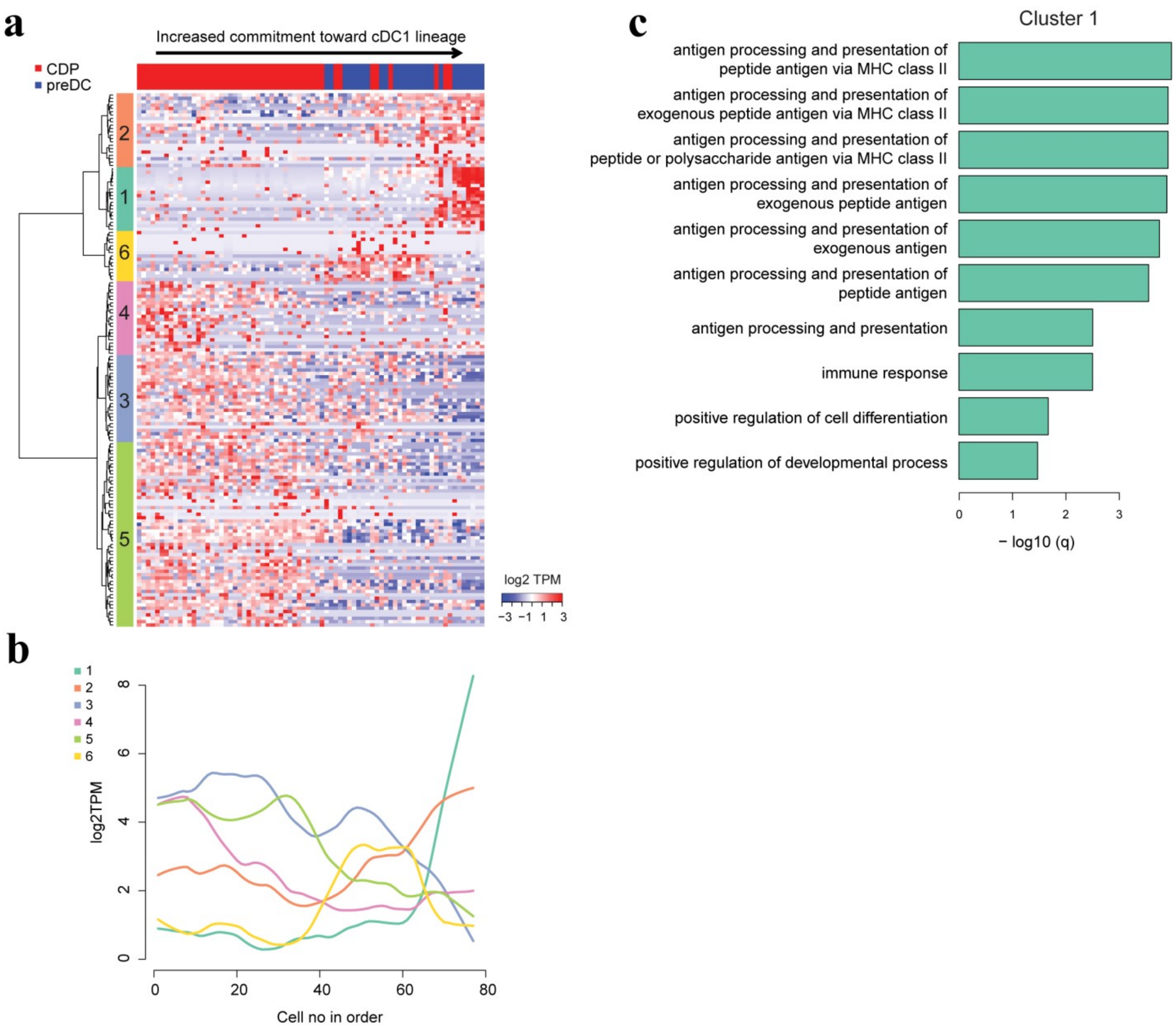

Supplementary Figure 3. Mpath re-ordered single cells and revealed sequential waves of gene regulation during cDC1 subset commitment.

Mpath re-ordered single cells along the developmental trajectory of cDC1 lineage commitment. (a) Heatmap and hierarchical clustering of genes that were differentially expressed along the developmental trajectory from CDP to cDC1-committed preDC. Cells were placed in the order of increased commitment toward cDC1 lineage. Clustering of genes identified six distinct groups. (b) Average expression per group was smoothed by loess regression and plotted in the line chart. (c) Gene ontology analysis identified biological processes that were significantly enriched by genes from group 1.

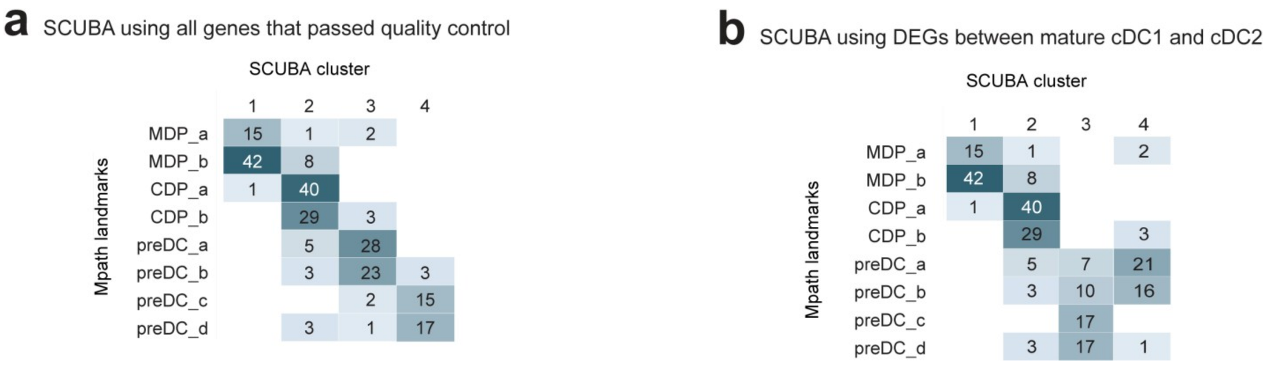

Supplementary Figure 4. Cross-table of cells from SCUBA clusters and Mpath landmark clusters.

We compared Mpath with SCUBA on the mouse DC progenitor dataset using all genes that passed quality control or DEGs between mature cDC1 and cDC2. SCUBA and Mpath generated different cluster sets. (a) Cross-table of cells from SCUBA clusters and Mpath landmark clusters when all genes that passed quality control were used. (b) Cross-table of cells from SCUBA clusters and Mpath landmark clusters when DEGs between mature cDC1 and cDC2 were used.

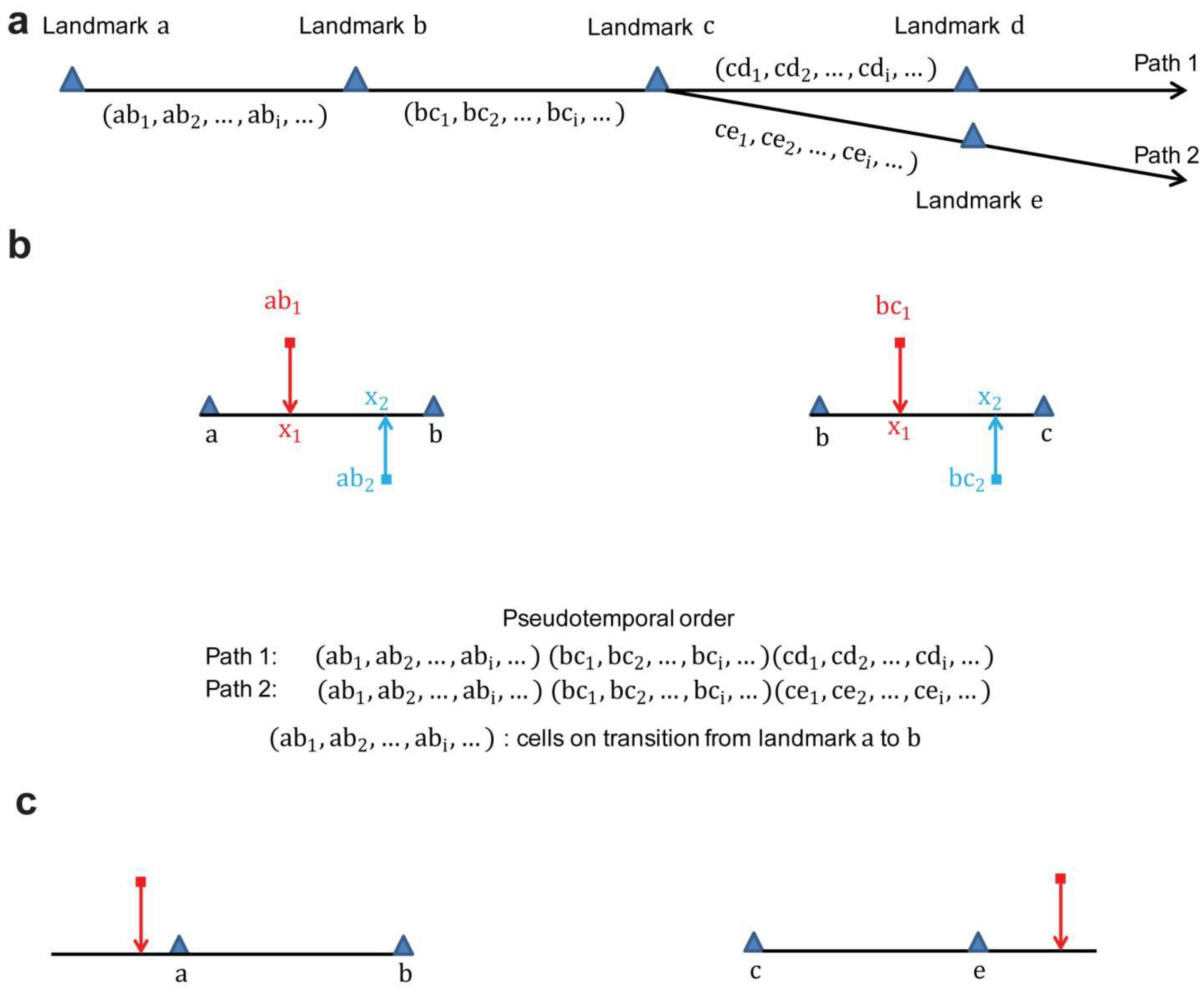

Supplementary Figure 5. Mpath re-ordered single cells along the multi-branching trajectories.

(a) Mpath computationally reconstructs cell developmental pathways as a multi-destination journey on a map of connected landmarks wherein individual cells are placed in order along the paths connecting the landmarks. (b) Mpath identifies cells that are potentially transitioning from landmark a to b based on their transcriptional proximities to both landmarks. To determine the ordering of these cells along the transition from landmark to , Mpath locates their projection points on the line spanning landmark and . For cell , Mpath draws two circles whose centers are at landmark and respectively, and whose radius are and respectively. It then projects the point where these two circles intersect onto the line connecting and , and identifies point . It repeats this process for cells and identifies their project points . Cells are then sorted according to the ordering of with respect to landmark and . (c) If a cell is “before” the first landmark or “after” the last landmark, we placed it on the extension of the edges connecting its two nearest landmarks.

Supplementary Tables

Supplementary Table 1. Gene ontology analysis identified biological processes enriched by genes up-regulated in landmark CDP_a compared to CDP_b.

Term	Genes	Benjamini
GO:0006974~response to DNA damage stimulus	RNF8, RPA1, KIF22, PGAP2, NEK1, BRE, BRIP1, RAD54L, CIB1	0.030611
GO:0000279~M phase	RNF8, RPA1, SPC25, CDC6, NEK1, ESPL1, BIRC5, BUB3, CDCA3	0.036964
GO:0007049~cell cycle	CDC6, MAEA, NEK1, PIM1, BIRC5, ESPL1, BANP, CDT1, RPA1, RNF8, SPC25, BUB3, CDCA3	0.042393
GO:0051276~chromosome organization	RNF8, RPA1, SMCHD1, BRE, HIST1H2AI, BANP, RBM14, RBBP7, RAD54L, BUB3, HIST2H2AA1	0.049673
