# Supplementary material for: Mpath maps multi-branching single-cell trajectories revealing progenitor cell progression during development
Source: Nat Commun. 2016 Jun 30;7:11988. doi: 10.1038/ncomms11988 (PMC4931327; doi:10.1038/ncomms11988)
Supplement: Supplementary Software — Mpath reference manual provides detailed documentation of Mpath R package. It covers how to install Mpath package, how to call Mpath functions and what are the expected outputs. It also includes example codes for performing Mpath analysis on the two datasets that were used in our manuscript. [file ncomms11988-s10.pdf]

Package ‘Mpath’

February 22, 2016

Type Package

Title Mpath: a single-cell RNAseq data analysis algorithm that maps multi-branching single-cell trajectories revealing cell progression during development.

Version 1.0

Date 2016-02-17

Author Dr Jinmiao CHEN

Maintainer Dr Jinmiao CHEN <chen_jinmiao@immunol.a-star.edu.sg>

Description This package provides a new single-cell RNAseq data analysis algorithm that maps multi-branching cell developmental pathways and aligns individual cells along the continuum of developmental trajectories. Mpath computationally reconstructs cell developmental pathways as a multi-destination journey on a map of connected landmarks wherein individual cells are placed in order along the paths connecting the landmarks. To achieve that, it first identifies clusters of cells and designates landmark clusters each defines a discrete cellular state. Subsequently it identifies and counts cells that are potentially transitioning from one landmark state to the next based on transcriptional similarities. It then uses the cell counts to infer putative transitions between landmark states giving rise to a state transition network. After that, Mpath sorts individual cells according to their various stages during transition to resemble the landmark-to-landmark continuum. Lastly, Mpath detects genes that were differentially expressed along the single-cell trajectories and identifies candidate regulatory markers.

License What license is it under?

Imports VGAM,
ggplot2,
gplots,
plyr,
reshape2

RoxygenNote 5.0.1

R topics documented:

Mpath-package 2
build_network 6
color_code_node_2 7
find_optimal_cluster_number 8
heatmap_nbor 9
landmark_designation 10
nbor_order 11
pseudotimePlotByGenes 12

QC_gene 13
SC_anova 13
SC_hc 14
SC_hc_colorCode 15
trim_net 16
vgam_deg 16
vgam_perGene 17

Index **18**

`Mpath-package` *Mpath: an analysis algorithm that maps multi-branching single-cell trajectories from single-cell RNA-sequencing data*

Description

This package provides a new algorithm that maps multi-branching cell developmental pathways and aligns individual cells along the continuum of developmental trajectories. Mpath computationally reconstructs cell developmental pathways as a multi-destination journey on a map of connected landmarks wherein individual cells are placed in order along the paths connecting the landmarks. To achieve that, it first identifies clusters of cells and designates landmark clusters each defines a discrete cellular state. Subsequently it identifies and counts cells that are potentially transitioning from one landmark state to the next based on transcriptional similarities. It then uses the cell counts to infer putative transitions between landmark states giving rise to a state transition network. After that, Mpath sorts individual cells according to their various stages during transition to resemble the landmark-to-landmark continuum. Lastly, Mpath detects genes that were differentially expressed along the single-cell trajectories and identifies candidate regulatory markers.

Examples

```
#### Install and load Mpath package

install.packages("Mpath_1.0.tar.gz",repos = NULL, type="source")
library(Mpath)

#################################################
###### Analysis of mouse DC dataset GSE60783 ####
#################################################

path <- getwd()
setwd(paste(path,"/GSE60783",sep=""))

##### remove low detection rate genes

rpkmFile="TPM_GSE60783_noOutlier.txt";
rpkmQCFile="TPM_GSE60783_noOutlier_geneQC0.05anyGroup.txt";
sampleFile="sample_GSE60783_noOutlier.txt";
QC_gene(rpkmFile=rpkmFile,
        rpkmQCFile=rpkmQCFile,
        sampleFile=sampleFile,threshold=0.05,method="any")

### Mpath using spleenic CD4 vs CD8 DEGs

rpkmFile = "TPM_GSE60783_noOutlier_geneQC0.05anyGroup_CD4vsCD8DEG.txt";
```

```
sampleFile = "sample_GSE60783_noOutlier.txt";
find_optimal_cluster_number(rpkmFile = rpkmFile,
                            sampleFile = sampleFile,
                            min_cluster_num = 7, max_cluster_num = 15,
                            diversity_cut = 0.6, size_cut = 0.05)

### Landmark designation

rpkmFile = "TPM_GSE60783_noOutlier_geneQC0.05anyGroup_CD4vsCD8DEG.txt";
baseName = "TPM_GSE60783_noOutlier_geneQC0.05anyGroup_CD4vsCD8DEG";
sampleFile = "sample_GSE60783_noOutlier.txt";
landmark_cluster <- landmark_designation(rpkmFile = rpkmFile,
                                         baseName = baseName,
                                         sampleFile = sampleFile,
                                         method = "diversity_size",
                                         numcluster = 11, diversity_cut=0.6,
                                         size_cut=0.05)

### Plot hierachical clustering

dataFile = "TPM_GSE60783_noOutlier_geneQC0.05anyGroup_CD4vsCD8DEG.txt";
SC_hc_colorCode(dataFile = dataFile,
                cuttree_k = 11,
                sampleFile= "sample_GSE60783_noOutlier.txt",
                width = 22, height = 10, iflog2 = TRUE,
                colorPalette = c("red","green","blue"))

### Construct weighted neighborhood network

exprs = "TPM_GSE60783_noOutlier_geneQC0.05anyGroup_CD4vsCD8DEG.txt";
baseName = "TPM_GSE60783_noOutlier_geneQC0.05anyGroup_CD4vsCD8DEG";
neighbor_network <- build_network(exprs = exprs,
                                  landmark_cluster = landmark_cluster,
                                  baseName = baseName)

### TrimNet: trim edges of lower weights

baseName = "TPM_GSE60783_noOutlier_geneQC0.05anyGroup_CD4vsCD8DEG";
trimmed_net <- trim_net(neighbor_network,textSize=30,
                        baseName = baseName,
                        method = "mst")

### plot trimmed net and color-code the nodes by gene expression

rpkmFile="TPM_GSE60783_noOutlier.txt";
lmFile="TPM_GSE60783_noOutlier_geneQC0.05anyGroup_CD4vsCD8DEG_landmark_cluster.txt";
color_code_node_2(networkFile=trimmed_net,
                  rpkmFile=rpkmFile,
                  lmFile=lmFile,
                  geneName=c("Irf8","Id2","Batf3"),
                  baseName="cDC1_marker",
                  seed=NULL)

### Re-order the cells on the path connecting landmark
### "CDP_2","CDP_1","PreDC_9","PreDC_3"

exprs = "TPM_GSE60783_noOutlier_geneQC0.05anyGroup_CD4vsCD8DEG.txt";
```

```
ccFile = "TPM_GSE60783_noOutlier_geneQC0.05anyGroup_CD4vsCD8DEG_landmark_cluster.txt";
order <- nbor_order(exprs = exprs,
                    ccFile = ccFile,
                    lm_order = c("CDP_2","CDP_1","preDC_9","preDC_3"),
                    if_bb_only=FALSE,
                    method=1)

### identify genes that changed along the cell re-ordering

deg <- vgam_deg(exprs = "TPM_GSE60783_noOutlier_geneQC0.05anyGroup.txt",
                order = order,
                lm_order = c("CDP_2","CDP_1","preDC_9","preDC_3"),
                min_expr=1,
                p_threshold=0.05)

### plot heatmap of genes that changed along the cell re-ordering

heatmap_nbor(exprs = "TPM_GSE60783_noOutlier_geneQC0.05anyGroup.txt",
             cell_order = "CDP_2_CDP_1_preDC_9_preDC_3_order.txt",
             plot_genes = "CDP_2_CDP_1_preDC_9_preDC_3_vgam_deg0.05.txt",
             cell_annotation = "sample_GSE60783_noOutlier.txt",
             num_gene_cluster = 6,
             hm_height = 15, hm_width = 10,
             baseName = "CDP_2_CDP_1_preDC_9_preDC_3_order_vgam_deg0.05")

###################################################
### Analysis of human myoblast dataset GSE52529 ###
###################################################

setwd(paste(path,"/GSE52529",sep=""))

### remove low detection rate genes

QC_gene(rpkmFile = "GSE52529_fpkm_matrix_nooutliers.txt",
        rpkmQCFile = "GSE52529_fpkm_matrix_nooutliers_geneQC0.05anyGroup.txt",
        sampleFile = "sample_nooutlier.txt",threshold=0.05,method="any")

rpkmFile = "GSE52529_fpkm_matrix_nooutliers_ANOVA_p0.05_DEG.txt";
sampleFile = "sample_nooutlier.txt";
find_optimal_cluster_number(rpkmFile = rpkmFile,
                            sampleFile = sampleFile,
                            min_cluster_num = 7, max_cluster_num = 18,
                            diversity_cut = 0.9, size_cut = 0.05)

rpkmFile = "GSE52529_fpkm_matrix_nooutliers_ANOVA_p0.05_DEG.txt";
baseName = "GSE52529_fpkm_matrix_nooutliers_ANOVA_p0.05_DEG";
landmark_cluster <- landmark_designation(rpkmFile = rpkmFile,
                                         baseName = baseName,
                                         sampleFile = "sample_nooutlier.txt",
                                         method = "diversity_size",
                                         numcluster = 14, diversity_cut=0.9,
                                         size_cut=0.05)

SC_hc_colorCode(dataFile = "GSE52529_fpkm_matrix_nooutliers_ANOVA_p0.05_DEG.txt",
                cuttree_k = 14,
                sampleFile= "sample_nooutlier.txt",
                width = 22, height = 10, iflog2 = TRUE)
```

```
### Construct weighted neighborhood network

exprs = "GSE52529_fpkm_matrix_nooutliers_ANOVA_p0.05_DEG.txt";
neighbor_network <- build_network(exprs = exprs,
                                  landmark_cluster = landmark_cluster)

### TrimNet: trim edges of lower weights

trimmed_net <- trim_net(neighbor_network,textSize=30,
                       baseName = "GSE52529_fpkm_matrix_nooutliers_ANOVA_p0.05_DEG",
                       method = "mst")

### Color code the landmarks with marker expression

networkFile="GSE52529_fpkm_matrix_nooutliers_ANOVA_p0.05_DEG_state_transition_mst.txt";
lmFile = "GSE52529_fpkm_matrix_nooutliers_ANOVA_p0.05_DEG_landmark_cluster.txt";
rpkmFile = "GSE52529_fpkm_matrix_nooutliers_geneSymbol.txt";
geneName=c("SPHK1","PBX1","XBP1","ZIC1","MZF1","CUX1","ARID5B","POU2F1","CDK1","MYOG");
color_code_node_2(networkFile = networkFile,
                  rpkmFile = rpkmFile,
                  lmFile = lmFile,
                  geneName = geneName,
                  baseName = "Marker",
                  seed=3)


### path 1: "T0_2","T0_1","T24_8","T48_10","T72_13"

ccFile = "GSE52529_fpkm_matrix_nooutliers_ANOVA_p0.05_DEG_landmark_cluster.txt";
order <- nbor_order(exprs = "GSE52529_fpkm_matrix_nooutliers_ANOVA_p0.05_DEG.txt",
                    ccFile = ccFile,
                    lm_order = c("T0_2","T0_1","T24_8","T48_10","T72_13"),
                    if_bb_only=TRUE,
                    method=1)

deg <- vgam_deg(exprs = "GSE52529_fpkm_matrix_nooutliers_geneQC0.05anyGroup.txt",
               order = order,
               lm_order = c("T0_2","T0_1","T24_8","T48_10","T72_13"),
               min_expr=1,
               p_threshold=0.05)

heatmap_nbor(exprs = "GSE52529_fpkm_matrix_nooutliers_geneQC0.05anyGroup.txt",
            cell_order = order,
            plot_genes = row.names(deg),
            cell_annotation = "sample_nooutlier.txt",
            num_gene_cluster = 6,
            hm_height = 15, hm_width = 10,
            baseName = "GSE52529_path1_order_vgam_deg0.05")

### path 2: "T0_2","T0_1","T24_7","T48_4","T72_14"
ccFile = "GSE52529_fpkm_matrix_nooutliers_ANOVA_p0.05_DEG_landmark_cluster.txt";
order <- nbor_order(exprs = "GSE52529_fpkm_matrix_nooutliers_ANOVA_p0.05_DEG.txt",
                    ccFile = ccFile,
                    lm_order = c("T0_2","T0_1","T24_7","T48_4","T72_14"),
                    if_bb_only = TRUE,
                    method=1)
```

```
deg <- vgam_deg(exprs = "GSE52529_fpkm_matrix_nooutliers_geneQC0.05anyGroup.txt",
               order = order,
               lm_order = c("T0_2","T0_1","T24_7","T48_4","T72_14"),
               min_expr=1,
               p_threshold=0.05)

heatmap_nbor(exprs = "GSE52529_fpkm_matrix_nooutliers_geneQC0.05anyGroup.txt",
            cell_order = order,
            plot_genes = row.names(deg),
            cell_annotation = "sample_nooutlier.txt",
            num_gene_cluster = 6,
            hm_height = 15, hm_width = 10,
            baseName = "GSE52529_path2_order_vgam_deg0.05")

### Generate Figure 5

ccFile = "GSE52529_fpkm_matrix_nooutliers_ANOVA_p0.05_DEG_landmark_cluster.txt";
order1 <- nbor_order(exprs = "GSE52529_fpkm_matrix_nooutliers_ANOVA_p0.05_DEG.txt",
                     ccFile = ccFile,
                     lm_order = c("T0_2","T0_1","T24_8","T48_10","T72_13"),
                     if_bb_only=TRUE,
                     method=1)

ccFile = "GSE52529_fpkm_matrix_nooutliers_ANOVA_p0.05_DEG_landmark_cluster.txt";
order2 <- nbor_order(exprs = "GSE52529_fpkm_matrix_nooutliers_ANOVA_p0.05_DEG.txt",
                     ccFile = ccFile,
                     lm_order = c("T0_2","T0_1","T24_7","T48_4","T72_14"),
                     if_bb_only=TRUE,
                     method=1)

deg1 <- read.table("T0_2_T0_1_T24_8_T48_10_T72_13_vgam_deg0.05.txt",sep="\t",header=T)
deg2 <- read.table("T0_2_T0_1_T24_7_T48_4_T72_14_vgam_deg0.05.txt",sep="\t",header=T)
deg <- unique(c(as.character(deg1[,1]),as.character(deg2[,1])))

heatmap_nbor(exprs = "GSE52529_fpkm_matrix_nooutliers_ANOVA_p0.05_DEG.txt",
            cell_order = c(order1,order2),
            plot_genes = deg,
            cell_annotation = "sample_nooutlier.txt",
            num_gene_cluster = 7,
            hm_height = 15, hm_width = 10,
            baseName = "Path12_method1orderedbackbone_progression_heatmap",
            n_linechart = list(order1,order2))
```

`build_network` *build_network constructs weighted neighborhood network*

Description

build_network constructs weighted neighborhood network

Usage

```
build_network(exprs, landmark_cluster, distMethod = "euclidean",
  baseName = NULL)
```

Arguments

`exprs:` a data frame or matrix of expression data(ie. rpkm, TPM, fpkm) containing cells in columns and genes in rows

`landmark_cluster:` a data frame or matrix of two columns or a tab delimited file of landmark cluster assignment of individual cells. The first column indicates cell ID, the second column indicates the landmark cluster which the cell was assigned to.

`dist_method:` the method for calculating dissimilarity between cells. distMethod can be one of "pearson", "kendall", "spearman" or "euclidean". Default is "euclidean".

Value

a matrix of weighted neighborhood network, column and row names are landmarks, the values represent the weights of the edges connecting two landmarks

Examples

```
exprs = "TPM_GSE60783_noOutlier_geneQC0.05anyGroup_CD4vsCD8DEG.txt";
baseName = "TPM_GSE60783_noOutlier_geneQC0.05anyGroup_CD4vsCD8DEG";
landmark = "TPM_GSE60783_noOutlier_geneQC0.05anyGroup_CD4vsCD8DEG_landmark_cluster.txt";
# or landmark can be the return value of landmark_designation function
neighbor_network <- build_network(exprs = exprs,
                                  landmark_cluster = landmark,
                                  baseName = baseName)
```

---

color_code_node_2 *color_code_node_2 plot state transition network in which nodes i.e. landmarks are color-coded by average expression of the given gene*

Description

color_code_node_2 plot state transition network in which nodes i.e. landmarks are color-coded by average expression of the given gene

Usage

```
color_code_node_2(networkFile, rpkmFile, lmFile, geneName, baseName = NULL,
  seed = NULL)
```

Arguments

`networkFile:` a tab delimited file containing a matrix of trimmed state transition network, column and row names are landmarks, the values are 0 or 1 indicating whether the two landmarks are connected.

`rpkmFile:` a tab delimited txt file of expression data, containing cells in columns and genes in rows

`lmFile:` a tab delimited file of landmark cluster assignment of individual cells. The first column indicates cell ID, the second column indicates the landmark cluster which the cell was assigned to.

`geneName:` gene name or a vector of gene names

`baseName:` prefix name of resulting files

Examples

```
rpkmFile="TPM_GSE60783_noOutlier.txt";
lmFile="TPM_GSE60783_noOutlier_geneQC0.05anyGroup_CD4vsCD8DEG_landmark_cluster.txt";
network="TPM_GSE60783_noOutlier_geneQC0.05anyGroup_CD4vsCD8DEG_state_transition_mst.txt";
# network can be the return value of trim_net
color_code_node_2(networkFile=network,
                  rpkmFile=rpkmFile,
                  lmFile=lmFile,
                  geneName=c("Irf8","Id2","Batf3"),
                  baseName="cDC1_marker",
                  seed=NULL)
```

---

find_optimal_cluster_number

find_optimal_cluster_number identifies the optimal number of initial cluster number by searching from min_cluster_num to max_cluster_num

---

Description

find_optimal_cluster_number identifies the optimal number of initial cluster number by searching from min_cluster_num to max_cluster_num

Usage

```
find_optimal_cluster_number(rpkmFile, sampleFile, min_cluster_num = 7,
  max_cluster_num = 13, diversity_cut = 0.6, size_cut = 0.05)
```

Arguments

`rpkmFile:` a tab delimited txt file of expression data, containing cells in columns and genes in rows

`sampleFile:` a tab delimited txt file of sample annotation with two columns, the first column is cell ID, the second column is group ID

`min_cluster_num:` minimum number of initial clusters

`max_cluster_num:` maximum number of initial clusters

`diversity_cut:` the cutoff value of diversity for differentiating landmark clusters from non-landmark clusters. The diversity of a landmark cluster must be below this cutoff.

`size_cut:` the cutoff value of size i.e. number of cells for differentiating landmark clusters from non-landmark clusters. The number of cells in a landmark cluster must be greater than this cutoff.

Examples

```
rpkmFile = "TPM_GSE60783_noOutlier_geneQC0.05anyGroup_CD4vsCD8DEG.txt";
sampleFile = "sample_GSE60783_noOutlier.txt";
find_optimal_cluster_number(rpkmFile = rpkmFile,
                            sampleFile = sampleFile,
                            min_cluster_num = 7, max_cluster_num = 15,
                            diversity_cut = 0.6, size_cut = 0.05)
```

`heatmap_nbor` *heatmap_nbor plot heatmap of gene expression*

Description

heatmap_nbor plot heatmap of gene expression

Usage

```
heatmap_nbor(exprs, cell_order, plot_genes, cell_annotation,
  num_gene_cluster = 4, hm_height = 10, hm_width = 10, baseName,
  colorPalette = NULL, n_linechart = NULL)
```

Arguments

`exprs` a data frame or matrix of expression data(ie. rpkm, TPM, fpkm) containing cells in columns and genes in rows

`cell_order` a vector storing the order of cells with cell ID or name, same as appeared in column names of `exprs`

`plot_genes` a vector storing the genes selected for plot, same as appeared in the row names of `exprs`

`cell_annotation` a two column data frame or matrix annotating cells(cell ID or name) with cell types

`num_gene_cluster` a integer indicating the number of gene clusters to generate by cutting the dendrogram tree, if num_gene_cluster = NULL, no gene clusters will be generated

`hm_height` an integeter to specify the heatmap height

`hm_width` an integeter to specify heatmap width

`baseName` a character string to specify the prefix of name of result files

`colorPalette` a character vector to specify the color scheme of column side bar that labels cell type of individual cells. Default value is NULL, a default color scheme will be deployed. Alternatively users can specfiy their desired color paletter, for examples, colorPalette=c("red","green","blue","black","orange","purple","burlywood4")

`n_linechart` a string vector to specify for which cell ordering to plot the line chart. Default value is NULL, all the cells in cell_order will be plot. Alternatively, users could specify, for examples two line charts, by n_linechart = list(c("cell1","cell2"),c("cell3","cell4"))

Examples

```
heatmap_nbor(exprs = "TPM_GSE60783_noOutlier_geneQC0.05anyGroup.txt",
             cell_order = "CDP_2_CDP_1_preDC_9_preDC_3_order.txt",
             plot_genes = "CDP_2_CDP_1_preDC_9_preDC_3_vgam_deg0.05.txt",
             cell_annotation = "sample_GSE60783_noOutlier.txt",
             num_gene_cluster = 6,
             hm_height = 15, hm_width = 10,
             baseName = "CDP_2_CDP_1_preDC_9_preDC_3_order_vgam_deg0.05")
```

---

landmark_designation

landmark_designation clusters cells and determines landmark clusters

---

Description

landmark_designation clusters cells and determines landmark clusters

Usage

```
landmark_designation(rpkmFile, baseName, sampleFile, distMethod = "euclidean",
  method = "kmeans", numcluster = NULL, diversity_cut = 0.6,
  size_cut = 0.05, saveRes = TRUE)
```

Arguments

`rpkmFile:`	a tab delimited txt file of expression data, containing cells in columns and genes in rows
`baseName:`	a character string indicating of prefix name of resulting files
`sampleFile:`	a tab delimited txt file of sample annotation with two columns, the first column is cell ID, the second column is group ID
`dist_method:`	the method for calculating dissimilarity between cells. distMethod can be one of "pearson", "kendall", "spearman" or "euclidean". Default is "euclidean".
`method:`	method for distinguishing landmark clusters from non-landmark clusters.method can be "kmeans" or "diversity" or "size" or "diversity_size". When method="diversity", numlm needs to be specified. Default is "diversity_size".
`numcluster:`	number of initial clusters
`diversity_cut:`	the cutoff value of diversity for differentiating landmark clusters from non-landmark clusters. The diversity of a landmark cluster must be below this cutoff.
`size_cut:`	the cutoff value of size i.e. number of cells for differentiating landmark clusters from non-landmark clusters. The number of cells in a landmark cluster must be greater than this cutoff.
`saveRes:`	a boolean to indicate whether to save result files

Value

a dataframe of two columns, the first column is cell ID, the second column is the landmark cluster the cell belongs to

Examples

```
rpkmFile = "TPM_GSE60783_noOutlier_geneQC0.05anyGroup_CD4vsCD8DEG.txt";
baseName = "TPM_GSE60783_noOutlier_geneQC0.05anyGroup_CD4vsCD8DEG";
sampleFile = "sample_GSE60783_noOutlier.txt";
landmark_cluster <- landmark_designation(rpkmFile = rpkmFile,
                                        baseName = baseName,
                                        sampleFile = sampleFile,
                                        method = "diversity_size",
                                        numcluster = 11, diversity_cut=0.6,
                                        size_cut=0.05)
```

---

nbor_order — *nbor_order sorts individual cells according to their various stages during transition to resemble the landmark-to-landmark continuum*

Description

nbor_order sorts individual cells according to their various stages during transition to resemble the landmark-to-landmark continuum

Usage

```
nbor_order(exprs, ccFile, lm_order = c("CD115+CDP_1", "PreDC_9", "PreDC_10",
  "PreDC_11"), if_bb_only = FALSE, method = 1)
```

Arguments

`exprs:`	a data frame or matrix of expression data(ie. rpkm, TPM, fpkm) or a tab delimited txt file of expression data, containing cells in columns and genes in rows
`ccFile:`	a data frame or matrix of two columns or a tab delimited file of landmark cluster assignment of individual cells. The first column indicates cell ID, the second column indicates the landmark cluster which the cell was assigned to.
`lm_order:`	a vector of landmark IDs indicating along which path the cells are to be sorted
`if_bb_only:`	a boolean to indicate if only cells on backbone will be sorted. Default is FALSE
`method:`	1 or 2 to indicate which method to be used for sorting. Default is 1

Value

a vector of re-orderd cell IDs

Examples

```
exprs = "TPM_GSE60783_noOutlier_geneQC0.05anyGroup_CD4vsCD8DEG.txt";
ccFile = "TPM_GSE60783_noOutlier_geneQC0.05anyGroup_CD4vsCD8DEG_landmark_cluster.txt";
order <- nbor_order(exprs = exprs,
                    ccFile = ccFile,
                    lm_order = c("CDP_2","CDP_1","preDC_9","preDC_3"),
                    if_bb_only=FALSE,
                    method=1)
```

`pseudotimePlotByGenes` *pseudotimeplotByGenes*

Description

pseudotimeplotByGenes

Usage

```
pseudotimePlotByGenes(exprs, if_log2 = TRUE, cell_annotation, cell_order,
  plot_genes = NULL, reverse_order = FALSE, min_expr = -3,
  cell_size = 2, plot_cols = NULL,
  trend_formula = "expression ~ sm.ns(Pseudotime, df=3)")
```

Arguments

- `exprs` a data frame or matrix of log transformed expression data, with row of cells and column of genes
- `cell_annotation` a two column data frame of matrix annotating cells(cell ID or name) with cell types
- `cell_order` a vector stroing the order of cells with cell ID or name, same as appeared in row names of `exprs`
- `plot_genes` a vector storing the genes selected for plot, same as appeared in the column names of `exprs`, if NULL, all genes in exprs will be selected
- `cell_size` the size of cells in the plot
- `trend_formula` the formula for regression analysis
- `min_exprs` the threshold for cutting of the cell expressions in regression values, values lower than this will be forced to `min_exprs`

Value

a object of ggplot

Examples

```
pseudotimePlotByGenes(exprs = "FULL.log2TPM.txt",
                      cell_annotation = "splAnnotation_outlierRemoved.txt",
                      cell_order = "uspin.PCA.0.03.seed1.txt",
                      plot_genes = "genes.txt",
                      reverse_order = TRUE, plot_cols = 3)
```

`QC_gene` *QC_gene removes genes that have TPM values < 1 in more than 95 percent of cells in each group*

Description

QC_gene removes genes that have TPM values < 1 in more than 95 percent of cells in each group

Usage

```
QC_gene(rpkmFile = "TPM_monocyte_Mar2015_noOutlier.txt",
  rpkmQCFile = "TPM_monocyte_Mar2015_noOutlier_geneQC0.05perGroup.txt",
  sampleFile = "monocyte_sample_Mar2015_noOutlier.txt", threshold = 0.05,
  method = "any")
```

Arguments

`rpkmFile`	a tab delimited file containing expression data (TPM, CPM, FPKM, etc), columns are cells and rows are genes.
`rpkmQCFile`	resulting file after QC
`sampleFile`	a tab delimited file containing sample annotations with two columns. The first column indicates SampleID, the second column indicates GroupID.
`threshold`	the cutoff of percentage of cells in which the given gene is not expressed.Default is 0.05.
`method`	keep genes whose rpkm values are not less than 1 in at least 5 percent of the cells in every group, method="any": keep genes whose rpkm values are not less than 1 in at least 5 percent of the cells in any group. Default is 'any'.

Examples

```
QC_gene(rpkmFile = "TPM_MDP_CDP_preDC_Mar2015_noOutlier.txt",
        rpkmQCFile = "TPM_MDP_CDP_preDC_Mar2015_noOutlier_geneQC0.05anyGroup.txt",
        sampleFile = "sample_MDP_CDP_preDC_Mar2015_noOutlier.txt",
        threshold = 0.05,method = "any")
```

`SC_anova` *SC_anova run ANOVA test to identify differentially expressed genes*

Description

SC_anova run ANOVA test to identify differentially expressed genes

Usage

```
SC_anova(inputfile, targetfile, iflog2, p_threshold, factor, baseName)
```

Arguments

`inputfile:` a tab delimied file containing expression values (TPM, CPM, FPKM, etc). Columns are cells and rows are genes.

`targetfile:` a tab delimited file indicating cell annoation with two columns. The first column indicates cell ID, the rest columns indicates cell annotations.

`iflog2:` a boolean value to indicate whether the expression values will be log2 transformed.

`p_threshold:` the cutoff of p values for DEGs.

`factor:` column name of targetfile, indicating which column will be used as cell annotation for comparison.

`baseName:` prefix name of resulting files.

Examples

```
inputfile="GSE52529_fpkm_matrix_nooutliers_geneQC0.05anyGroup.txt";
targetfile="GSE52529_fpkm_matrix_nooutliers_ANOVA_p0.05_DEG_landmark_cluster.txt";
baseName="GSE52529_fpkm_matrix_nooutliers_ANOVA_p0.05_DEG_landmark_cluster";
SC_anova(inputfile = inputfile,
         targetfile = targetfile,
         iflog2 = TRUE,p_threshold=0.05,factor="landmark_cluster",
         baseName = baseName)
```

`SC_hc` *SC_hc hierachical clustering of single cells*

Description

SC_hc hierachical clustering of single cells

Usage

```
SC_hc(dataFile, baseName, cuttree_k = 4)
```

Arguments

`dataFile:` a tab delimited txt file of expression data, columns are cells, rows are genes.

`baseName:` prefix name of resulting files

`cuttree_k:` number of clusters to be generated by cutting the dendrogram.

`SC_hc_colorCode` *SC_hc_colorCode hierarchical clustering of single cells, leaves color-coded by cell type information.*

Description

SC_hc_colorCode hierarchical clustering of single cells, leaves color-coded by cell type information.

Usage

```
SC_hc_colorCode(dataFile, baseName = NULL, cuttree_k = NULL,
  sampleFile = NULL, width = 22, height = 10, iflog2 = TRUE,
  colorPalette = NULL, distMethod = "Euclidean")
```

Arguments

- `dataFile:` a tab delimited txt file of expression data, columns are cells, rows are genes.
- `baseName:` prefix name of resulting files
- `cuttree_k:` number of clusters to be generated by cutting the dendrogram. cuttree_k can be an integer or NULL. If NULL, no clusters will be generated.
- `sampleFile:` a tab delimited txt file of cell annotation, the first column is cell ID, the second column is group ID.
- `width:` width of the resulting figure
- `height:` height of the resulting figure
- `iflog2:` a boolean value indiciating if the expression data will be log2 transformed.
- `colorPalette:` if NULL, brewer.pal(n,"Set2") will be used, or colorPalette can be c("red","green","blue").
- `distMethod:` distMethod can be one of "pearson", "kendall", "spearman" or "Euclidean"

Examples

```
dataFile = "TPM_GSE60783_noOutlier_geneQC0.05anyGroup_CD4vsCD8DEG.txt";
SC_hc_colorCode(dataFile = dataFile,
                cuttree_k = 11,
                sampleFile= "sample_GSE60783_noOutlier.txt",
                width = 22, height = 10, iflog2 = TRUE,
                colorPalette = c("red","green","blue"))
```

`trim_net` *trim_net trimms the weighted neighborhood network by removing edges of lower weights*

Description

trim_net trimms the weighted neighborhood network by removing edges of lower weights

Usage

```
trim_net(nb12, textSize = 20, baseName = NULL, method = "mst",
  start = "MDP_6")
```

Arguments

`nb12:`	a matrix of weighted neighborhood network, column and row names are landmarks, the values represent the weights of the edges connecting two landmarks
`baseName:`	a character string indicating the prefix name of resulting files
`method:`	trimming method, method can be one of "TrimNet" or "mst". When method="TrimNet" the initial node needs to be specified. Default is "mst"
`start:`	starting landmark, needs to be specified when method="TrimNet".

Value

a matrix of trimmed state transition network, column and row names are landmarks, the values are 0 or 1 indicating whether the two landmarks are connected.

Examples

```
baseName = "TPM_GSE60783_noOutlier_geneQC0.05anyGroup_CD4vsCD8DEG";
trimmed_net <- trim_net(neighbor_network,textSize=30,
                        baseName = baseName,
                        method = "mst")
```

`vgam_deg` *vgam_deg identifies genes that were differentially expressed along the re-ordered single-cell trajectories using vgam*

Description

vgam_deg identifies genes that were differentially expressed along the re-ordered single-cell trajectories using vgam

Usage

```
vgam_deg(exprs = "TPM_GSE60783_noOutlier_geneQC0.05anyGroup.txt", order,
  lm_order = c("CD115+CDP_2", "CD115+CDP_1", "PreDC_9", "PreDC_3"),
  min_expr = 1, p_threshold = 0.05)
```

Arguments

`exprs:`	a data frame or matrix of expression data(ie. rpkm, TPM, fpkm) or a tab delimited txt file of expression data, containing cells in columns and genes in rows
`order:`	a vector of re-ordered cell IDs
`lm_order:`	a vector of landmark IDs indicating along which path the cells are to be sorted
`min_expr:`	a numeric value indicating the minimum TPM value for a gene to be considered as expressed. Default is 1.
`p_threshold:`	p value cutoff for selecting differentially expressed genes.

Value

deg: a list of differentially expressed genes

Examples

```
deg <- vgam_deg(exprs = "TPM_GSE60783_noOutlier_geneQC0.05anyGroup.txt",
                order = order,
                lm_order = c("CDP_2","CDP_1","preDC_9","preDC_3"),
                min_expr=1,
                p_threshold=0.05)
```

`vgam_perGene` *vgam_perGene determines if a gene is differentially expressed along the re-ordered single-cell trajectories using vgam*

Description

vgam_perGene determines if a gene is differentially expressed along the re-ordered single-cell trajectories using vgam

Usage

```
vgam_perGene(expr, order, min_expr)
```

Arguments

`expr:`	a vector of one gene's expression in different cells (ie. rpkm, TPM, fpkm)
`order:`	a vector of re-ordered cell IDs
`min_expr:`	a numeric value indicating the minimum TPM value for a gene to be considered as expressed. Default is 1.

Value

pval: p value of significance of the gene being differentially expressed

Examples

```
p_val <- vgam_perGene(expr,order,min_expr=1)
```

Index

build_network, 6

color_code_node_2, 7

find_optimal_cluster_number, 8

heatmap_nbor, 9

landmark_designation, 10

Mpath-package, 2

nbor_order, 11

pseudotimePlotByGenes, 12

QC_gene, 13

SC_anova, 13
SC_hc, 14
SC_hc_colorCode, 15

trim_net, 16

vgam_deg, 16
vgam_perGene, 17
